# Probing the Dynamics of Low-Overpotential CO_2_-to-CO Activation on Copper Electrodes with Time-Resolved Raman Spectroscopy

**DOI:** 10.1021/jacs.2c03172

**Published:** 2022-08-11

**Authors:** Jim de Ruiter, Hongyu An, Longfei Wu, Zamorano Gijsberg, Shuang Yang, Thomas Hartman, Bert M. Weckhuysen, Ward van der Stam

**Affiliations:** Inorganic Chemistry and Catalysis, Debye Institute for Nanomaterials Science, Utrecht University, Universiteitsweg 99, 3584 CG Utrecht, The Netherlands

## Abstract

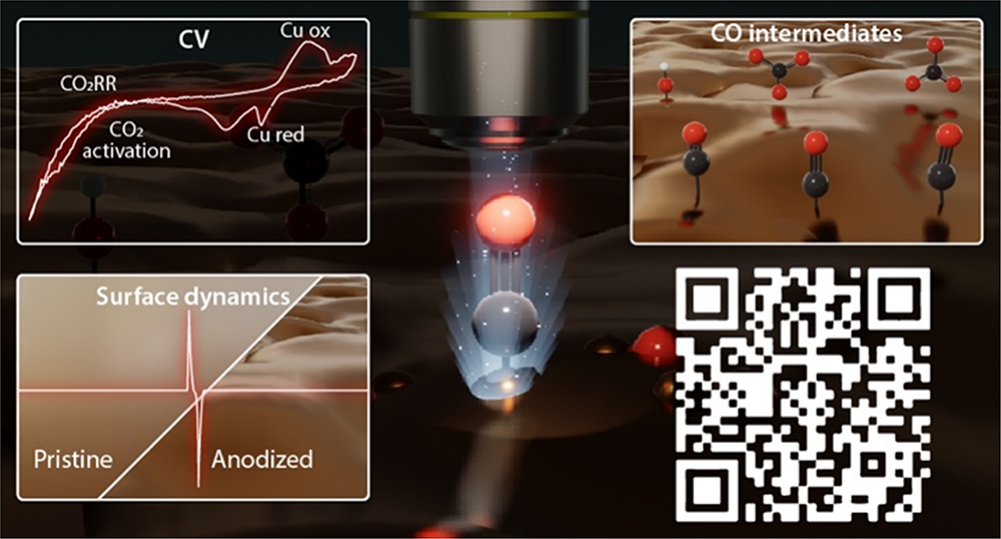

Oxide-derived copper electrodes have displayed a boost in activity and selectivity toward valuable base chemicals in the electrochemical carbon dioxide reduction reaction (CO2RR), but the exact interplay between the dynamic restructuring of copper oxide electrodes and their activity and selectivity is not fully understood. In this work, we have utilized time-resolved surface-enhanced Raman spectroscopy (TR-SERS) to study the dynamic restructuring of the copper (oxide) electrode surface and the adsorption of reaction intermediates during cyclic voltammetry (CV) and pulsed electrolysis (PE). By coupling the electrochemical data to the spectral features in TR-SERS, we study the dynamic activation of and reactions on the electrode surface and find that CO_2_ is already activated to carbon monoxide (CO) during PE (10% Faradaic efficiency, 1% under static applied potential) at low overpotentials (−0.35 V_RHE_). PE at varying cathodic bias on different timescales revealed that stochastic CO is dominant directly after the cathodic bias onset, whereas no CO intermediates were observed after prolonged application of low overpotentials. An increase in cathodic bias (−0.55 V_RHE_) resulted in the formation of static adsorbed CO intermediates, while the overall contribution of stochastic CO decreased. We attribute the low-overpotential CO_2_-to-CO activation to a combination of selective Cu(111) facet exposure, partially oxidized surfaces during PE, and the formation of copper-carbonate-hydroxide complex intermediates during the anodic pulses. This work sheds light on the restructuring of oxide-derived copper electrodes and low-overpotential CO formation and highlights the power of the combination of electrochemistry and time-resolved vibrational spectroscopy to elucidate CO2RR mechanisms.

## Introduction

The utilization of renewably generated electricity to convert carbon dioxide (CO_2_) into fuels and base chemicals is of fundamental and technological interest.^1−3^ Copper stands out as an electrode material due to its unique ability to reduce CO_2_ into hydrocarbon products, yielding a variety of (valuable) C_1_,^4^ C_2_,^5,6^ and C_3_ hydrocarbons.^7,8^ However, the large overpotentials and low selectivity for multi-carbon products still hampers the large-scale implementation of CO_2_ electrolyzers. The structure and morphology of the catalytic surface, as well as the composition of the electrolyte, are key factors that determine the activity and selectivity of the CO_2_ reduction reaction (CO2RR).^9−17^ Understanding the interplay between the surface structure, the electrolyte, and the intermediates under reaction conditions is therefore important to steer the CO_2_ reduction reaction to the desired product with high selectivity at low overpotential. Recent experimental and theoretical work has uncovered the importance of positively charged copper species (Cu^+^ and Cu^δ+^) to tune the selectivity of copper electrocatalysts toward C_2_ products.^16,18−23^ These positively charged copper species are a result of alternating oxidation/reduction cycles in cyclic voltammetry (CV, anodic treatment) or pulsed electrolysis (PE) experiments. Moreover, lower CO2RR overpotentials were observed for copper oxide-derived catalysts^16,18−23^ in PE experiments,^24−30^ but the mechanism behind the increased selectivity and reduction of overpotential by positively charged copper species is still debated.

In order to correlate the dynamic surface structure and the presence of positively charged copper species during PE and CV to the increased catalyst selectivity and activity, both the structure and the reaction need to be probed on the same time scale. For this purpose, time-resolved surface-enhanced Raman spectroscopy (TR-SERS) is a great analytical tool^31^ since it allows the study of the copper surface (Raman shifts < 700 cm^–1^), the ions (>1000 cm^–1^), and reaction intermediate species, such as CO (>2000 cm^–1^), with sub-second time resolution.

Here, we perform TR-SERS during both CV and PE on oxide-derived copper electrodes to link the time- and potential-dependent restructuring of the catalyst surface to the observed reduction/oxidation features in CV and PE as well as the reaction intermediates at the electrode surface. Through the combination of electrochemistry and TR-SERS, we find that the activation of the electrode surface through oxide removal (∼0.3 V_RHE_) is immediately followed by the approach of bicarbonate and carbonate electrolyte ions close to the electrode surface. When a sufficiently large cathodic bias is applied (−0.65 V_RHE_), vibrations corresponding to linear adsorbed CO at copper (step-edge) sites (at 280, 360, and 2090 cm^–1^) dominate the TR-SERS data in agreement with the literature.^15,20,32−37^ However, dynamic vibrational features associated with adsorbed CO on an activated copper surface (between 2000 and 2100 cm^–1^) already appear in the combined CV and TR-SERS experiments at a moderate cathodic bias of −0.35 V_RHE_. This low-overpotential CO_2_ activation to gaseous CO was confirmed by combined PE and TR-SERS experiments, which revealed stochastic CO vibrations and a Faradaic efficiency (FE) of 10% CO for PE at −0.35 V_RHE_ (compared to 1% CO FE at fixed cathodic bias). Ex situ surface-sensitive X-ray diffraction (XRD) measurements revealed the dynamic restructuring of the electrode surface during CV and PE experiments, resulting in a Cu(111) dominant surface after PE and a Cu(100) dominant surface after CV. This restructuring in combination with the partially oxidized surface during PE is inferred to result in low-overpotential CO_2_ activation and subsequent CO formation. The time- and potential-dependent spectral features during the combined PE and TR-SERS experiments revealed that not only does the catalyst surface change, the carbonate electrolyte ions near the surface also form complex structures with depleted oxidized copper species. Raman features in the carbonate region show similar dynamic behavior directly after each anodic pulse, suggesting that they are correlated to the Raman features in the CO region. The combination of these stochastic CO and carbonate vibrations strongly suggests that anodic pulses induce an alternative route to CO that proceeds via a copper-carbonate-hydroxide intermediate, which is in line with recent literature.^38−41^ The results obtained highlight the importance of time-resolved spectroscopic studies to couple the anodization of the surface CO2RR to surface changes and reveal the interplay between the electrode surface, electrolyte ions, and reaction intermediates.

## Results and Discussion

### Combined Cyclic Voltammetry and Time-Resolved Raman Spectroscopy

Time-resolved surface-enhanced Raman spectroscopy (TR-SERS) has been used to study an electrodeposited copper (CuED) electrode during cyclic voltammetry (CV) (Supporting Information, Figure S1a) in a 0.1 M CO_2_-saturated potassium bicarbonate (KHCO_3_) electrolyte solution at pH = 6.8 with the aim to couple the structure of the copper electrode surface to the adsorbed species as a function of both potential and time. The first cycle of the CV scan starts at +0.55 V_RHE_ (open circuit potential, OCP) and proceeds in the cathodic direction to −0.85 V_RHE_, after which the scan is reversed in the anodic direction up to +1.05 V_RHE_ (step size, 10 mV; scan rate, 10 mV/s). After anodic treatment at +1.05 V_RHE_, the scan direction is reversed again and the subsequent cycles (cycle 2–4, Figure S1a) are very repeatable. Several oxidation and reduction features can be observed in the CV scans, which are ascribed to the oxidation and reduction of the electrode surface (between +1.05 and −0.2 V_RHE_) as well as the onset of CO2RR and the hydrogen evolution reaction (HER) (below −0.4 V_RHE_). The broad reduction wave in the first cycle is ascribed to Cu_2–*x*_O layer removal, which originates from contact with aqueous electrolyte solutions during the electrodeposition procedure.^23^

**Figure 1:**
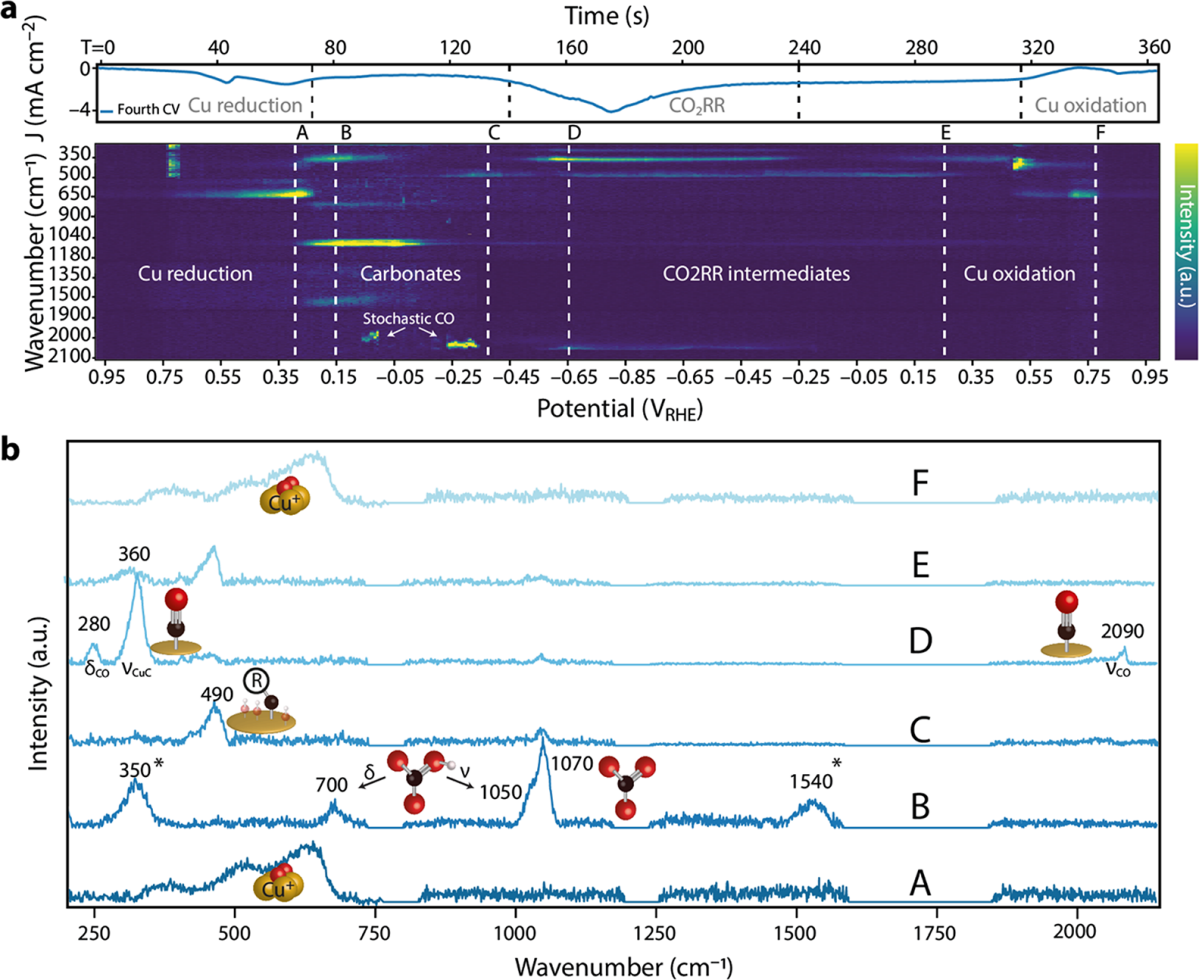
(a) Time-resolved surface-enhanced Raman spectroscopy (TR-SERS) data taken on the surface of electrodeposited copper (CuED) during cyclic voltammetry (CV) as function of time (upper *x* axis) and potential (bottom *x* axis). The heatmap represents the baseline-corrected Raman intensity. The induced current of the CV measurement is illustrated above the heatmap to correlate CV features to the Raman spectroscopy signals. Detailed information on the construction of these data is provided in the main text. More data can be found in Figure S3. (b) 2D Raman plots of specific moments in time corresponding to the dashed lines in (a). The peaks with asterisks (*) are still under debate in the literature, which is explained in the main text. To ensure fast spectral collection (1 s per spectrum), the measurements were conducted in static mode in which data was collected in small Raman windows while omitting other Raman windows, resulting in the flat lines in (b). A more detailed explanation is provided in the Experimental Section in the SI.

After the anodic bias, two sharper reduction bands are observed at +0.45 V and +0.25 V, which are tentatively ascribed to CuO and Cu_2–*x*_O reduction, respectively, based on literature.^42,43^ The pristine CuED electrode and the electrode after CV treatment were analyzed with ex situ scanning electron microscopy (SEM) (Figures S1b and S2) and surface-sensitive grazing incidence X-ray diffraction (XRD) measurements (Figure S1c), which revealed restructuring of the electrode surface facets but showed conservation of the morphology. As can be seen in Figure S1c, the pristine CuED electrodes consisted of metallic Cu and Cu_2–*x*_O domains, but after CV treatment and CO2RR, the surface primarily consists of metallic copper. The absence of copper oxide reflections in these ex situ experiments suggests that the electrodes are not reoxidized during sample transfer and air exposure. The dendrites of the pristine CuED are still clearly present after the CV treatment, and the preferred orientation of Cu(100) facets is observed, in line with literature.^44^ In order to unambiguously assign the observed reduction and oxidation waves in the CV scans to the dynamic restructuring of the electrode surface, TR-SERS experiments with a time resolution of one spectrum per second were performed (Figure 1a).

In Figure 1a, the TR-SERS spectra are presented in a heatmap, where the *y* axis represents the Raman wavenumbers and the double *x* axes were used to present the data as function of time (top axis) and potential (bottom axis) during the fourth CV scan, with the Raman scattering intensity plotted in the *z* direction and illustrated using the heat map (viridis).The combined CV and TR-SERS data for the first three CV cycles can be found in Figure S3a.

The top part of Figure 1a shows the current as a function of applied potential plotted on top of the TR-SERS data, which enables us to link the CV waves to the observed changes in TR-SERS. Furthermore, line spectra at selected points during the CV scan are plotted in Figure 1b for clarity, which were collected at points A and B in Figure 1a. The CV data can be roughly divided into three parts: (1) Cu reduction (+0.55 to −0.45 V), (2) CO2RR/HER (<−0.45 V), and (3) Cu oxidation (>+0.55 V). The TR-SERS spectra can also be roughly divided into three parts: (1) <700 cm^–1^: Cu_2–*x*_O, Cu-C, and Cu-O(H) vibrations; (2) 700–1600 cm^–1^: carbonate/bicarbonate electrolyte ions; and (3) 2000–2100 cm^–1^: adsorbed CO stretching vibrations. The potential-dependent spectral features were studied in more detail with TR-SERS. During an anodic sweep, copper is oxidized and barely any vibrations are observed due to lack of the SERS-active Cu surface. However, upon reversing the scan direction after a maximum anodic bias of +1.0 V_RHE_, a reducing current is observed in the CV scan around +0.7 V_RHE_. At the same time, vibrations appear at ∼630, 520, and 400 cm^–1^, which are assigned to surface Cu_2–*x*_O according to the literature.^45,46^ These bands are ascribed to (partially) Raman-active modes of the Cu_2–*x*_O lattice (520 cm^–1^, T_2g_) as well as the rich defect chemistry of copper oxides (400 and 630 cm^–1^). Changes in the relative ratio of these bands as a function of applied potential are inferred to indicate variations in the defect chemistry (Figure S4).^45,46^ Furthermore, in Figure 1a, it is observed that the Cu_2–*x*_O Raman vibrations grow in intensity as a function of time and applied potential, suggesting that the signals are enhanced more by the underlying Cu metal electrode, and hence, the oxide layer becomes thinner. This can be explained by the local field enhancement of the underlying metallic copper: when the nanostructured Cu_2–*x*_O surface of the electrode gets more reduced, the local field enhancement rises with it, resulting in strong Raman signal enhancement of vibrations of the thin surface Cu_2–*x*_O layer. This is further confirmed by the abrupt disappearance of the Cu_2–*x*_O bands in the TR-SERS data after the maximum of the second reduction band in the CV scan (+0.25 V_RHE_). This indicates that the surface is completely reduced to metallic Cu^0^, leaving behind an activated surface ready for CO2RR.

### Bicarbonate/Carbonate Electrolyte Species

After the reduction and activation of the surface to Cu^0^, the immediate approach of electrolyte ions close to the electrode is observed, evidenced by the stretching vibrations at 1070 and 1050 cm^–1^ corresponding to carbonate (CO_3_^2–^) and bicarbonate (HCO_3_^–^) electrolyte ions, respectively.^47−49^ Although the electrolyte consists of bicarbonate (HCO_3_^–^) ions, carbonate (CO_3_^2–^) ions are observed as well immediately after the surface oxide layer is removed. This is caused by the increase in local alkalinity through the formation of hydroxide (OH^–^) near the surface^50−53^ during surface oxide removal, which results in the formation of CO_3_^2–^ species through rapid deprotonation of the HCO_3_^–^ electrolyte ions from the neutral pH (6.8) electrolyte solution. Since the 1070 cm^–1^ vibrational feature is almost identical to the vibrational mode of carbonate ions in solution (see Figure S5), it is implied that the carbonate ions are not directly bound to the surface but most probably close enough to be enhanced by the copper surface. It is noted that the SERS effect of species close to a partially oxidized electrode surface is weak, and therefore, electrolyte species (e.g., (bi)carbonate ions) cannot be discerned before the surface is completely activated to metallic Cu^0^, which happens around a cathodic bias of 0.25 V_RHE_ according to the TR-SERS data (Figure 1a).

Next to the vibrations at 1050 and 1070 cm^–1^, bands at 1540, 700, and 350 cm^–1^ rise simultaneously (Figure 1a,b). The origin of these bands are still under debate in the literature: the band at 350 cm^–1^ is assigned to the carboxylate adsorption of the first reduction intermediate of CO_2_,^35^ but recent literature showed that this band could correspond to the adsorbed bidentate carbonate species to which the 1540 cm^–1^ band can also be assigned.^54^ In our time-resolved SERS measurements, we observe that these bands (350 and 1540 cm^–1^) appear and disappear synchronously in time and applied potential, indicating that these bands are related. We can see that these bands are only present in a relatively short potential window (+0.25 and −0.20 V_RHE_,), redshift (∼20 cm^–1^; Figure S3c) with increasing cathodic bias (associated with the electrochemical Stark effect),^35,37^ and disappear before the onset potential of CO2RR (−0.4 V_RHE_); see Figure S3c. As the adsorption of the carboxylate intermediate (η_2_(C,O)-CO_2_^–^)^35^ is typically considered to react further to CO, it can be debated whether these bands can be assigned to the carboxylate intermediate. Since these bands appear simultaneously in the same potential window as the carbonate species, it is more likely to assign these bands to adsorbed carbonate species. Furthermore, in two recent papers, carbon-13 labeling was performed, which showed contradicting results. In the work of Moradzaman et al.,^34^ this band does not change by increased carbon mass, where in the work of Chernyshova et al.,^35^ a redshift was observed. As both theories in literature have strong scientific arguments, we leave the exact assignment open. Finally, we observe a band at 700 cm^–1^. According to the fundamental vibrational modes of carbonate, the bending mode of carbonates is expected to give a band at ∼700 cm^–1^.^55^ We therefore assign the band at ∼700 cm^–1^ to the bending mode of carbonate ions in solution since it appears and disappears in the same potential window as the symmetric stretching vibration of carbonate at 1070 cm^–1^ (+0.25 to −0.25 V_RHE_).

### Observation of Surface Copper–Oxygen and Copper–Carbon Species

Around −0.15 V_RHE_, the intensity of the 1050 and 1070 cm^–1^ bands decreases, while the bands at 350 and 700 cm^–1^ disappear. Subsequently, broad bands at ∼495 and ∼ 440 cm^–1^ appear. An accurate assignment of these Raman vibrations is highly complex as various species can be found in this spectral region, for example, Cu^0^-OH,^56−58^ Cu-C, and Cu-O.^47,54^ At a higher cathodic bias (−0.45 to −0.85 and back to −0.25 V_RHE_, CO2RR window) the band at 445 cm^–1^ shifts to 460 cm^–1^, while the band at 495 cm^–1^ remains at its position (Figure 1). Recent ^13^CO_2_ labeling experiments suggested that a Cu-C species causes a vibration at 502 cm^–1^, which is often accompanied with shoulder bands at ∼440–525 cm^–1^, and we tentatively ascribe the observed vibrational features at 445 and 460 cm^–1^ to similar species (denoted as Cu-C-R hereafter).^34^ Around −0.55 V_RHE_, vibrations at 2090, 360, and 280 cm^–1^ appear simultaneously. These vibrations are typically assigned to the linear CO stretching, Cu-C stretching, and Cu-CO bending of adsorbed (linear) CO on Cu, respectively.^20,36,47,59^ Recently, Roldan-Cuenya et al. have shown that these vibrations can be used as a probe to measure the surface coverage of CO by taking the ratio of the two low-Raman shift vibrations (280 and 360 cm^–1^),^20^ which is also possible through onstream substitution of the reactant isotope.^60^ In the potential window of −0.55 to −0.85 V_RHE_, it is observed that the high-frequency-band (HFB) CO band at 2090 cm^–1^ dominates and has a low-frequency-band (LFB) CO tail centered around 2050 cm^–1^. These potential- and time-dependent CO stretching vibrations are attributed to the stochastic behavior of adsorbed CO and are extensively described in our previous work, where we studied the time-dependent behavior of adsorbed CO at fixed cathodic biases.^31^ However, the stochastic behavior of adsorbed CO on the CuED electrode is also clearly visible in the present CV experiments, where vibrations at different Raman shifts between 2070 and 2095 cm^–1^ can be found in the potential window from −0.55 to −0.85 V_RHE_ in the forward scan. Furthermore, we observed hysteresis in CO adsorption and desorption in the forward and backward scan, respectively (see Figure S6), similar to the work of Waegele et al.^36^ If we take the maxima of the CO vibrations between 2089 and 2096 cm^–1^ in this potential window and plot the average vibrational energy against the potential, a hysteresis profile for CO adsorption/desorption is obtained. It is observed that in each cycle, the intensity of the CO_ad_ in the forward scan (−0.2 to −0.85 V_RHE_, purple line) maximizes around −0.7 V_RHE_, whereas in the backward scan, the maximum CO_ad_ is observed at −0.35 V_RHE_. This indicates that at these low overpotentials after electrode activation, CO is still adsorbed/produced at the electrode surface.

**Scheme 1:**
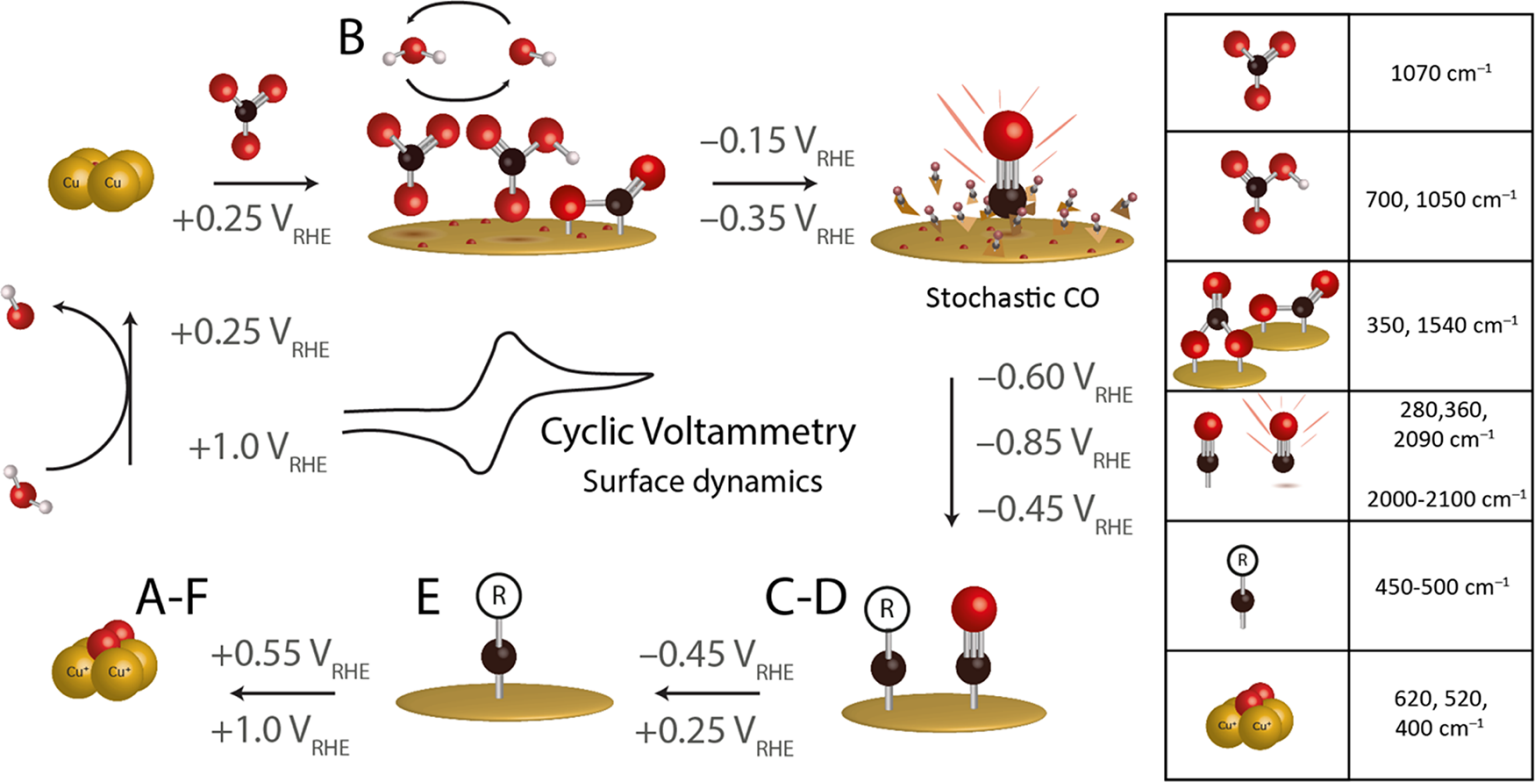
Schematic Overview of Surface Dynamics during Cyclic Voltammetry (CV) on Electrodeposited Cu as Observed with Time-Resolved Surface-Enhanced Raman Spectroscopy (TR-SERS) The vibrations of the proposed surface species are shown in the right panel, and the schematics and the corresponding potential windows in which these species are present on or near the surface are depicted in the arrows (above and below the arrow are the start and end potentials, respectively). The letters A–F refer to the spectra in Figure 1b in which the surface species can be observed with TR-SERS. An overview of all vibrations with more detail is provided in Table S1.

Scheme 1 summarizes the events that occur in a concerted manner on the electrode surface during a CV scan. The CV measurement can be roughly divided in four regions: reduction of copper oxides (+0.70 to +0.30 V_RHE_), oxidation of copper (>0.55 V_RHE_), CO_2_RR intermediates (< −0.45 V_RHE_), and adsorption/coordination of electrolyte species in the low-overpotential region (+0.25 to −0.45 V_RHE_). If we take a closer look in the low-overpotential region in the TR-SERS heatmap in Figure 1a, stochastic CO stretching vibrations are already present in the forward scan (cathodic direction) around −0.30 V_RHE_. These low-overpotential Cu-CO vibrations are not observed at a fixed wavenumber, and their intensity fluctuates as a function of time, highlighting the stochastic nature of these vibrations. This is in contrast to the linear CO vibrations observed at higher cathodic bias (−0.6 to −0.85 V_RHE_), which consistently appear at 2050 and 2090 cm^–1^. The energy of the CO stretching vibration depends on the adsorption geometry at the surface. For example, bridged CO is found at lower wavenumbers (1900–2000 cm^–1^) compared to linearly adsorbed CO (2000–2100 cm^–1^), since the electron density is more on the C, which weakens the C-O vibrational energy.^31^ Furthermore, the exposed Cu facet also influences the vibrational energy.^61^ For example, linear CO adsorbed on Cu(100) gives rise to a band at 2045 cm^–1^,^62^ but on (110) terrace sites, a band at 2055 cm^–1^ is observed.^37,63^ We therefore hypothesize that in the low-overpotential window of 0 to −0.4 V, the copper surface is dynamically rearranging, thereby exposing different Cu facets and oxidation states that give rise to a wide distribution of CO vibrational energies, hereafter referred to as stochastic CO vibrations.

### Structural Rearrangements after Cyclic Voltammetry and during Pulsed Electrolysis

**Figure 2:**
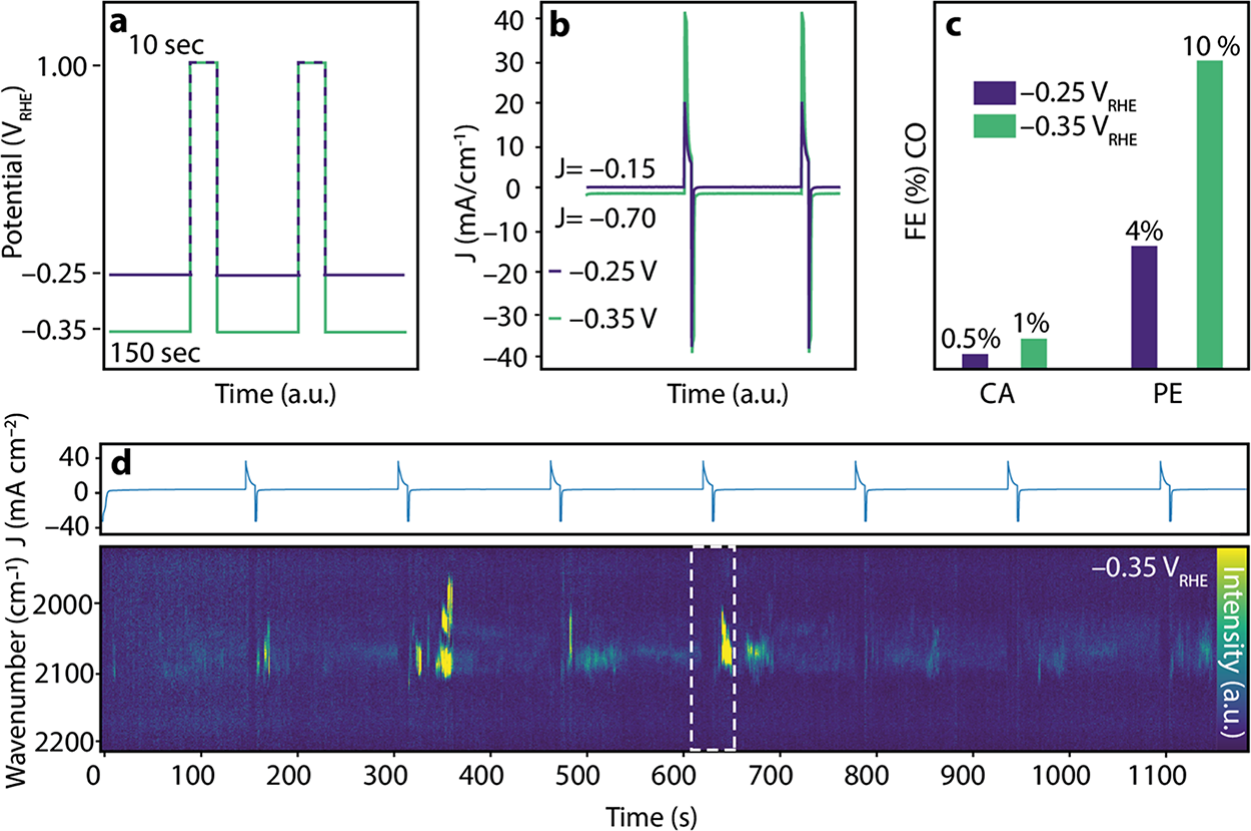
Overview of the time-resolved surface-enhanced Raman spectroscopy (TR-SERS) data taken on the surface of electrodeposited copper (CuED) during pulsed electrolysis (PE) experiments. (a) Schematic representation of the programs used in the PE experiments. (b) Current vs time traces obtained by pulsed electrolysis at −0.25 and −0.35 V_RHE_ for 150 s alternated by 10 s of +1.0 V_RHE_. (c) Averaged Faradaic efficiencies to gaseous CO in chronoamperometry (CA) and PE experiments at −0.25 and −0.35 V_RHE_. Corresponding partial current densities for CO and H_2_ can be found in Figure S8. (d) TR-SERS in the Cu-CO spectral window (1950–2200 cm^–1^) during PE at −0.35 V_RHE_. Intensity is plotted in a heatmap as a function of time. The PE data is positioned above the heatmap to overlap with the Raman data. The area indicated with the white dashed lines is shown in more detail in Figure 3.

**Figure 3:**
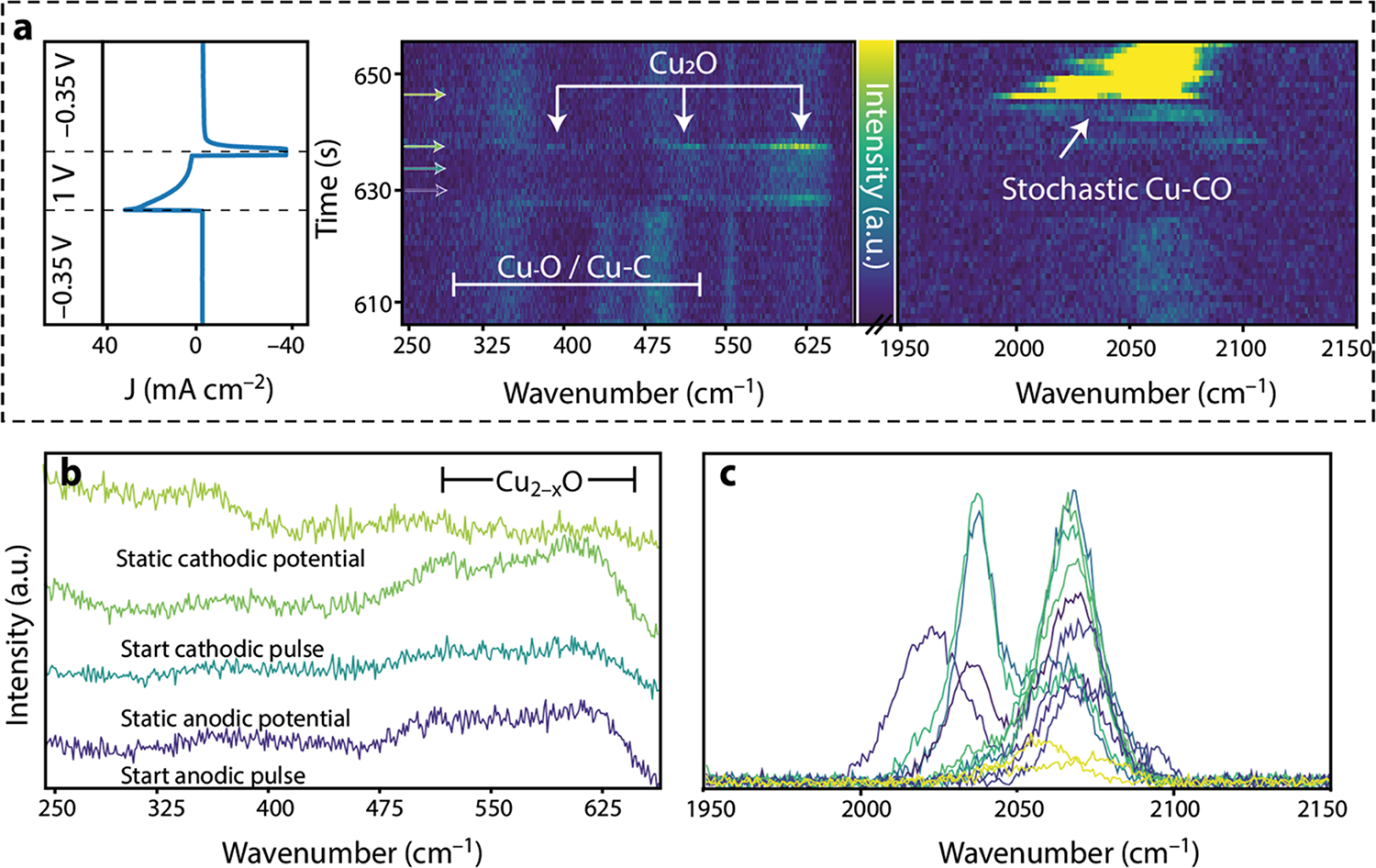
TR-SERS and PE data at low and high Raman shift. (a) Zoom-in of the events that occur during one pulse of Figure 2d (indicated by the dashed box), showing Cu_2–*x*_O formation during the anodic pulse and stochastic CO formation after the cathodic bias is applied again, focusing on the Cu-C/Cu-O and Cu-CO spectral windows (i.e., 250–650 and 1950–2150 cm^–1^). (b, c) 2D TR-SERS plots of specific moments in time in the (b) low Raman window and (c) CO Raman window, respectively, corresponding to areas indicated by the colored arrows in the heatmap in (a).

To further study how the Cu surface is dynamically changing in the low-overpotential window, we performed pulsed electrolysis (PE) experiments to mimic the cycling between oxidation and reduction conditions in the CV scans and combined the PE experiments with in situ TR-SERS (Figures 2 and 3) as well as ex situ grazing incidence X-ray diffraction (GI-XRD) measurements (Figure S7). The PE experiments were performed at a fixed anodic potential (+1.05 V_RHE_) and varying cathodic potentials (e.g., −0.25 and – 0.35 V_RHE_). Cathodic pulses of 150 s were alternated by 10 s anodic pulses as illustrated in Figure 2a,b.

Ex situ GI-XRD measurements were carried out to analyze the restructuring of the electrode surface due to PE and anodic treatment. In Figure S7b, the GI-XRD patterns before and after CV and PE are displayed. The surface of the pristine CuED electrode consists of a mixture of Cu_2–*x*_O and Cu due to the inevitable exposure of the Cu electrode to air and water during the electrodeposition procedure. By analyzing the area ratio between the (111) and (200) reflections at diffraction angles of 43 and 52°, respectively, the restructuring of the electrode surface as a result of the electrochemical treatment can be elucidated. In the pristine electrode, the (111)/(200) ratio is 0.95. After the CV treatment of the CuED electrode, the Cu(111)/Cu(200) ratio is changed to 0.44, meaning that the surface of the electrode is Cu(100)-dominant. We note that no copper oxide reflections can be discerned, suggesting that air exposure and sample transfer does not result in severe oxidation of the electrodes. From the scanning electron microscopy (SEM) images in Figure S7a (more SEM images can be found in Figure S2), it is evident that the morphology of the electrode surface has hardly changed. However, the relatively harsh oxidative potentials during the anodic treatment of the CV scan could have dissolved some copper ions, which are later redeposited under cathodic potentials and have preferentially formed Cu(100) facets, as also discussed in the literature.^44,64^ After applying a potential of −0.35 V_RHE_ for 2 h, the Cu(111)/Cu(200) ratio is 0.88, which is a similar ratio as for the pristine CuED. This observation indicates that the electrode surface is quite stable under moderate applied potentials, although the surface of the dendrites is smoothened. We find that the Cu(111)/Cu(200) ratio has drastically changed to 2.9 after PE experiments at −0.35 V_RHE_, which is ascribed to the constant switching between the oxidation and reduction of the surface. The latter also resulted in strong morphology changes, as observed in the SEM image in Figure S7a. The dendrites appear to be crumbled into smaller nanostructures, which primarily consisted of the Cu(111) phase based on the GI-XRD measurements. According to literature, Cu(111) surfaces are more active toward C_1_ products, such as methane and CO.^12,65^ In order to analyze the effect of the dynamic restructuring of the electrode surface to a Cu(111)-dominant surface during pulsed electrolysis to the performance of the electrodes, we performed activity and selectivity measurements.

### Low-Overpotential Pulsed Electrolysis and Time-Resolved Surface-Enhanced Raman Spectroscopy

The activity and selectivity of the PE experiments were measured using an electrochemical H-cell connected to an (online) GC. According to literature, the activity of a copper foil electrode toward carbon-containing products is very low in the low-overpotential region (<−0.4 V_RHE_), and primarily, HER occurs.^11,12^

The activity and selectivity were analyzed by performing chronoamperometry (CA) measurements with and without anodic pulses (+1.0 V_RHE_) at cathodic biases of −0.35 and −0.25 V_RHE_. It can be noted in Figure 2c that the Faradaic efficiency toward CO is very low in the absence of anodic pulses. At a static applied potential of −0.35 V_RHE_, an average FE for CO of ∼1% is observed, which reduces over time as the electrocatalyst deactivates. At an applied potential of −0.25 V_RHE_, the production of CO is almost negligible, and it was only in the first two or three injections (∼ 15 min) that some CO was detected, which roughly corresponded to 0.5% FE for CO. It appears that the pristine CuED material contains active sites that can induce the formation of CO at such low overpotentials, but prolonged exposure of the electrode to reducing potentials deactivates the catalyst. The GI-XRD measurements described above showed that the ratio between exposed Cu(111) and Cu(100) surfaces of the electrode is unaltered by these low overpotentials (Figure S7). In literature, this initial activity at low overpotentials is usually ascribed to sub-surface oxygen on oxide-derived copper electrodes.^18−21,66^

When the low-overpotential cathodic bias is alternated with the anodic pulses, a drastic change in CO FE is observed compared to the CA measurements (Figure 2c). The electrode performance is stable over the course of 5.5 h and the CO FE is boosted to 4% at −0.25 V_RHE_, and 10% at −0.35 V_RHE_, compared to the static application of the same potential (Figure 2c). The increased production of CO during PE is potentially related to the selective exposure of Cu(111) under these conditions (Figure S7). The corresponding partial current densities of the PE and CA experiments can be found in Figures S8 and S9. It is observed that the catalyst becomes slightly more active over time, as can be seen in Figures S10a,b and S11a,b. At −0.25 V_RHE_, the FE toward CO is initially 3%; after 5.5 h, it increased to 4%, and at −0.35 V_RHE_, the FE for CO increased from 10 to 12% over the course of 5.5 h (Figures S10 and S11). This observation could be explained by an increase in the electrochemical active surface area (ECSA) before and after the PE experiments (Figure S10c,d and S11c,d). We have analyzed the ECSA before and after PE, which showed that the roughness factor increased by 1.35 and 1.13 at −0.25 and – 0.35 V_RHE_, respectively. The increase in surface roughness is ascribed to the iterative oxidation of the copper surface and redeposition of copper ions to the surface. However, this increase in surface roughness cannot solely explain the boosted activity, which we ascribe to partially oxidized surfaces and preferential exposure of Cu(111) due to PE (Scheme 1). By combining the PE experiments with TR-SERS measurements, we can elucidate what is happening on the surface of the CuED during PE (at −0.35 V_RHE_). As shown in Figures 2d and 3a, many Cu-CO species with different vibrational energies are present in the TR-SERS spectra in the 2000–2100 cm^–1^ wavenumber region, similar to the combined CV and TR-SERS measurements at low overpotentials. We attribute the spread of CO vibrational peak positions to a mixture of Cu facets and partially oxidized surface sites. In this view, the adsorbed CO acts as a probe that visualizes the rearrangement of the surface induced by the constant oxidation and reduction of the surface during PE. This is also in line with our ex situ GI-XRD data and shows that the introduction of the anodic pulses results in severe rearrangement of the surface, which forms highly active catalytic sites that can activate CO_2_ at low overpotentials. It is evident that primarily low-frequency CO bands (2000–2050 cm^–1^) are present and that the intensity of these bands is most intense in the first 10–30 s after each anodic pulse (Figure 3a). Similar stochastic behavior of adsorbed CO can be observed at lower cathodic potentials (−0.35 to −0.05 V_RHE_; Figure S12). This suggests that most of the Cu-CO is formed on the freshly reduced copper surface, which indicates that the activity of the Cu surface is the highest directly after each anodic pulse (i.e., oxidation). We exclude the possibility that the high intensity of the low-overpotential “stochastic CO” is solely a SERS enhancement effect since the activity measurements during the PE experiments showed that the observed Cu-CO intermediate at low overpotentials did result in the production of gaseous CO (Figure 2c). We therefore infer that the observed increase in CO signal intensity in TR-SERS (Figure 2d) is a consequence of both a higher production of CO combined with a local increase in SERS due to surface roughening after switching from anodic to cathodic bias and that the observed trends in “stochastic CO” intensity and enhanced CO production during low-overpotential PE are interlinked. This is further corroborated by the combination of PE and TR-SERS experiments alternated with CA and TR-SERS (Figure S9). These experiments unambiguously showed that gaseous CO production dropped to 2–3 FE% in the absence of pulses and recovered to 10 FE% during PE, while “stochastic CO” was abundantly present during PE and absent during CA.

In Figure 3 and Figure S13, it can be seen that before the anodic pulse, Cu-O and Cu-C vibrations (<600 cm^–1^) are also present (Figure 3a) besides the stochastic linear CO vibrations at around 2000–2100 cm^–1^ (Figure 3c). Furthermore, it is observed that Cu_2–*x*_O is formed instantly when an anodic bias is applied, as evidenced by the presence of the corresponding Cu_2–*x*_O Raman bands (Figure 3b). During the anodic pulse, the Cu-O and Cu-C vibrations are not visible anymore, suggesting that the anodic current has resulted in desorption of the adsorbed intermediates. When the cathodic bias is applied again, the spectra are almost identical as prior to the pulse, but very intense and stochastic signals associated with Cu-CO are observed, as can be seen in Figure 3c, without any additional line broadening. Furthermore, the Raman band at ∼620 cm^–1^ is weakly present after the onset of the cathodic bias, which indicates that Cu_2–*x*_O could still be present on the surface under reducing conditions at early time scales after the anodic pulse. The other characteristic bands for Cu_2–*x*_O (at ∼520 and ∼400 cm^–1^) could not be discerned. Recent DFT calculations have shown that surface oxygen can be stable up to −0.84 V_RHE_,^18^ which is in line with our combined TR-SERS and PE experiments. The results for the PE experiments in the low and high wavenumber region have shown that (1) the “stochastic CO” is repeatedly dominant directly after switching from the anodic to cathodic pulse (i.e., the appearance of this vibration is reproducible), but the intensity fades over time; (2) the wavenumber and intensity during the evolution of “stochastic CO” under cathodic bias vary between pulses, highlighting the stochastic nature of this vibration; and (3) no additional line broadening is observed for the “stochastic CO” vibrations.

### Potential-Dependent TR-SERS and the Presence of Stochastic CO

**Figure 4:**
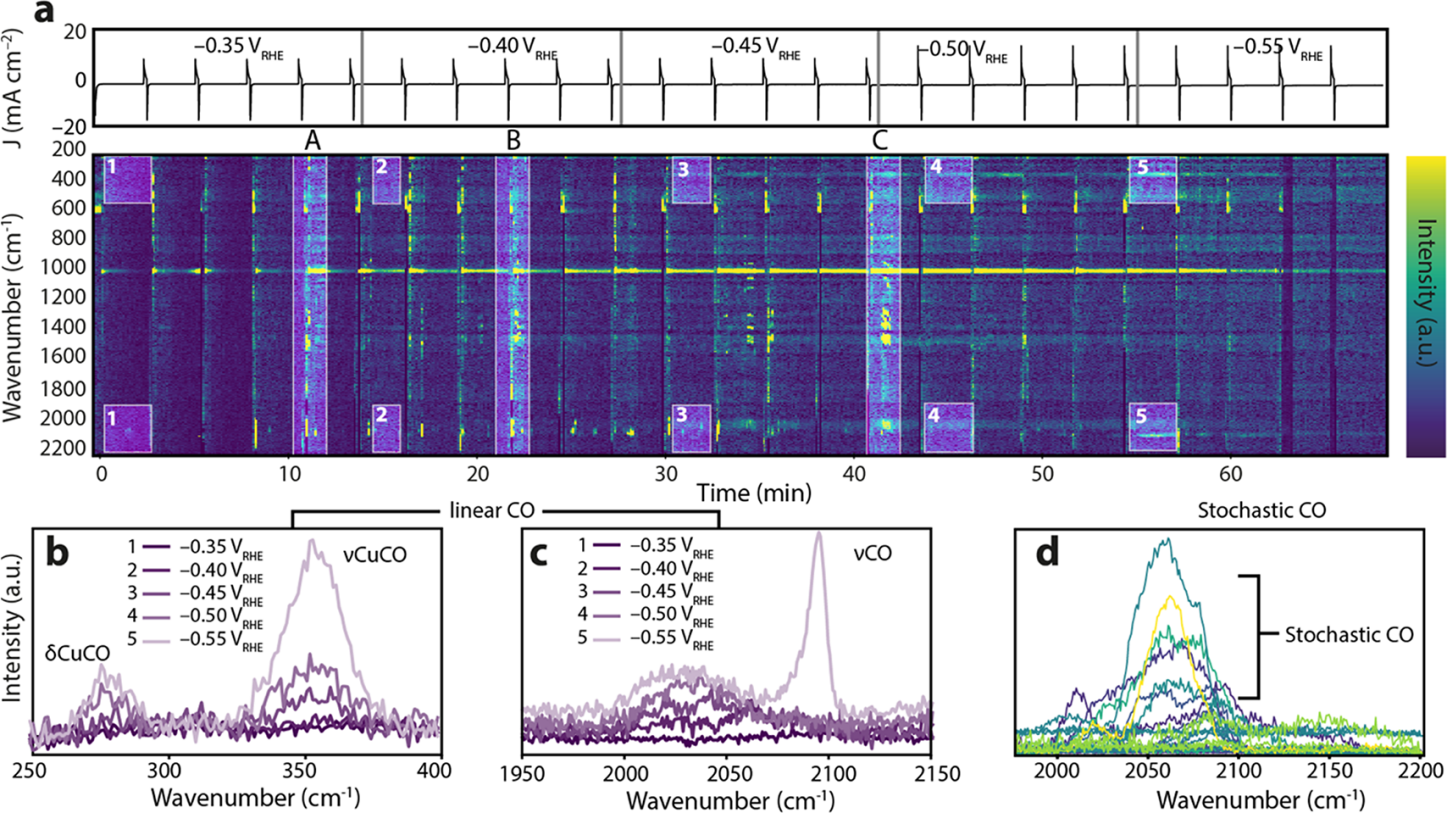
Pulsed electrolysis (PE) experiment on CuED using 150 s of cathodic pulses and 10 s of anodic pulses at different applied cathodic biases. The anodic pulse was always set to +1.0 V_RHE_, and the cathodic pulse was subsequentially increased after five pulses by 50 mV, obtaining a sequence of −0.35, −0.40, −0.45, −0.50, and – 0.55 V_RHE_. (a) The heatmap shows the Raman spectra collected over time. The *x* axis indicates the time, and the *y* axis corresponds to the wavenumber. The colors of the heatmap show the (baseline-corrected) intensity. The top graph shows the current profile over time and the change in cathodic potential. (b) Averaged baseline-corrected spectra of the conventional δCuCO (275 cm^–1^) and νCuCO (360 cm^–1^) at each potential at timescales of 10–150 s of the cathodic pulse. Averages were taken in the highlighted regions in the heatmap, indicated by 1–5. (c) Same as (b), but in the CO region. (d) All spectra in the νCO region showing high intensities for the stochastic CO vibrations, which can also be seen in the heatmap as high-intensity spots between 2000–2150 cm^–1^ directly after the anodic pulses.

To further understand the difference in the stochastic CO during the reconstruction of the surface and the more static CO vibrations at higher cathodic bias observed in the CV and PE measurements, we performed a pulse program where we subsequently increased the cathodic potential while keeping the 10 s anodic pulse at +1.0 V_RHE_. In this way, we could study the formation of the CO intermediate during an (increasing) cathodic static potential at long time scales after the cathodic pulse onset as well as the formation of the stochastic CO directly after the cathodic pulse onset. The number of pulses per cathodic potential was set at 5 to minimize the total measurement time but still get sufficient statistics during consecutive anodic pulses to investigate the influence of the pulses on the catalytic behavior. Figure 4 shows the combined PE and in situ Raman spectroscopy experiment at varying cathodic biases between −0.35 and 0.55 V_RHE_ (with steps of 50 mV) for 150 s alternated with a +1.0 V_RHE_ pulse for 10 s. The activity data for this experiment can be found in Figure S14.

During each anodic pulse, the Cu_2–*x*_O Raman signals at 400, 520, and 630 cm^–1^ can clearly be observed in the low-Raman shift region (Figure 4a). When the cathodic potential is applied again, the signal disappears within a few seconds, indicating that the Cu_2–*x*_O phase at the surface is readily removed. We note that residual (sub)surface oxides can still be present, but they are undetectable by Raman spectroscopy due to their low concentration. When the Raman spectroscopy data at early times after the anodic pulse is compared to the spectra in the tail of the cathodic pulse, clear differences can be observed in the CO region (∼2000 cm^–1^), which become more evident when the cathodic potential is increased (see Figure 4a–c). The introduction of anodic pulses seems to have no influence on the formation of the *CO intermediates on long timescales after the anodic pulse at a sufficient cathodic bias (Figure 4b,c). Upon application of a cathodic bias of −0.45 V, the characteristic Cu-CO bending, Cu-C linear stretching, and the stretching modes of low-frequency and high-frequency band linear CO are visible (at 280, 360, and 2050/2090 cm^–1^, respectively).^20,36,47,59^ At a lower cathodic bias, e.g., −0.35 V_RHE_, these bands are absent at longer timescales after the cathodic pulse, indicating that CO_2_ is not activated at these potentials under static conditions. In Figure 4b,c, line plots are shown that correspond to the averaged spectra collected at the five different cathodic potentials in the tail of the cathodic pulse. The regions in which the averaged spectra were calculated are highlighted in the heatmap and tagged “1” to “5”. Regardless of the oxidative pulses at early times, the CO bands show the characteristic behavior, according to the existing literature, of the negative Stark shift of the 360 cm^–1^ band upon increasing the cathodic potential and the formation of a sharp CO band at 2090 cm^–1^ (high-frequency band CO) at −0.55 V_RHE_.^20,36,47,59^

The stochastic CO vibrations that were described in the previous section are also clearly present in these PE experiments at varying cathodic biases (Figure 4d). What is clear from our experiments is that the stochastic CO is only observed on (pre)oxidized copper surfaces. In the previous work of Roldan Cuenya et al., a similar Raman signal in the CO region was observed at low overpotentials on copper oxide-derived catalysts.^20^ The authors referred to studies that indicated that this signal originates from hydrogen atoms on the copper surface (e.g., Cu-H). However, we have performed isotope labeling experiments with deuterated water, which unambiguously showed that these vibrations are not influenced by the relative high mass of ^2^H and thus cannot be ascribed to H on the copper surface (Figure S15). If H was involved in the observed reaction pathway, giving rise to adsorbed Cu-H species with vibrational energy around 2050 cm^–1^, a substantial decrease in the vibrational energy is expected for Cu-D (Figure S15). Since we do not observe stochastic vibrations in this spectral region, it is suggested that there should be an alternative explanation for the existence of the stochastic Raman signals at around 2000 cm^–1^. To confirm whether the observed vibrations around 2000 cm^–1^ are indeed from adsorbed CO during PE, experiments with ^13^CO_2_ were performed, which displayed clear shifts to lower wavenumbers as expected due to the increase in mass between ^12^CO_2_ and ^13^CO_2_ (Figure S16).

### Stochastic (Bi)Carbonate Vibrations and Its Correlation with CO Formation

It is important to mention that in our Raman spectroscopy experiments, we do not simultaneously observe the bands for stochastic CO and the bands at low Raman shift (ascribed to νCu-C and δCuCO).^20^ This strongly suggests that these stochastic vibrations originate from different reaction intermediates with different symmetries compared to linear Cu-CO, resulting in different active Raman modes. This is analogous to the absence of Cu-C vibrations in the low-Raman shift region for bridged CO, which also has a different symmetry compared to linear CO on a Cu surface. On the other hand, our correlated PE and TR-SERS data in Figure 4 reveal stochastic vibrations in the carbonate region (1000–1700 cm^–1^) that coincide with the stochastic CO vibrations directly after the anodic pulse, suggesting that the anodic pulses have significant influence on the (bi)carbonate system that is present close to the electrode–electrolyte interface. The carbonate symmetrical stretch vibration at 1065 cm^–1^ is present during the whole experiment regardless of cathodic bias. Next to these spectator carbonate ions, higher intensities between 1200 and 1600 cm^–1^ are visible directly after the pulse (Figure 4 and Figure S17). Similar to the stochastic CO, the stochastic carbonate signals appear in a non-ordered manner and differ significantly compared to the averaged spectra at longer timescales (Figure S17).

In Figure S17, zoom-in line plots are presented in the carbonate and CO regions at different cathodic bias, corresponding to the highlighted regions “A” to “C” in Figure 4a. It is evident that at low overpotentials (−0.35 V_RHE_), multiple vibrations can be discerned directly after the oxidation pulse, whereas no vibrations are observed after applying the cathodic bias for a longer time both in the carbonate region and the CO region. The entire spectral range of these data sets, as well as more spectra, can be found in Figure S18.

An overview of wavenumbers for all known species in the carbonate–bicarbonate–CO_2_ system is given in Table S1.^33,35,47−49,55,67,68^ Many species in this spectral region give rise to vibrations that mainly involve (symmetrical) stretching vibrations of C–O bonds in the carbonate molecules. The observation of these stochastic vibrations in the carbonate region, together with the appearance of the stochastic vibrations of CO species, suggests that carbonates are directly involved in the reduction toward CO and are connected to the anodization of the surface. We performed PE in different bicarbonate electrolyte concentrations (0.05 to 1.0 M) to investigate whether the stochastic vibrations of both CO and carbonate depend on the carbonate concentration and local alkalinity (Figure S19). All experiments showed stochastic vibrations in both the carbonate and CO spectral window, suggesting that the variation in local alkalinity does not influence the boosted activity of the partially oxidized surface due to PE.^24^

Furthermore, we observed the formation of small precipitates in the electrolyte when pulsed electrolysis was performed for several hours. Analysis of these precipitates with Raman spectroscopy revealed the vibrational footprint of a malachite phase (i.e., Cu_2_CO_3_(OH)_2_),^68^ as can be seen in Figure S20. The formation of such complex copper carbonate hydroxide salts was already postulated in earlier work on Cu-catalyzed CO_2_ electroreduction, where complex copper salts are formed as surface layers in CV measurements.^69^ Recently, Jiang et al. showed with Raman spectroscopy that copper carbonate hydroxide can act as an intermediate to produce CO,^38^ in which malachite is directly reduced to CO. This hypothesis is in line with the high-intensity signals we observed in the carbonate and CO Raman regions as well as the boosted activity for CO at low overpotentials (see Figure S21). Furthermore, copper carbonate transient species were elucidated through in situ fluorescence spectroscopy, and the authors invoked these transient copper carbonate complexes as highly active phases for CO formation in a dissolution–redeposition mechanism, in line with our findings.^70^ We therefore ascribe the low-overpotential CO_2_-to-CO activation observed in our combined PE and TR-SERS experiments to an alternative reaction pathway that involves malachite copper carbonate hydroxide complexes in the electrolyte solution, which generates a highly active site upon redeposition and subsequent reduction.

## Conclusions

In this work, we have utilized time-resolved surface-enhanced Raman spectroscopy (TR-SERS) during cyclic voltammetry (CV) and pulsed electrolysis (PE) to elucidate the restructuring of the electrode surface and its influence on the performance of the electrode in CO_2_ reduction reactions (CO2RR) over copper at relatively low overpotentials (−0.35 V_RHE_). The observed oxidation and reduction features in the CV measurements were coupled to the time- and potential-dependent vibrational features of surface and near-surface species, and the activation of the oxidized copper electrode surface for CO2RR was studied. Through the combination of these TR-SERS and CV experiments, the CV scan could be divided into four distinct regions: reduction of copper oxides (+0.70 to +0.30 V_RHE_), oxidation of copper (>0.55 V_RHE_), adsorption/coordination of electrolyte ions (+0.3 V to −0.45 V_RHE_), and CO_2_RR (< −0.45 V_RHE_). In the CO2RR region, the Raman signals associated with linear CO on Cu were observed between 200–500 and 2050–2100 cm^–1^. However, before the CO2RR onset at −0.45 V_RHE_, stochastic CO vibrations were already observed at low overpotentials (0 to −0.4 V_RHE_). The repetitive cycling between anodic and cathodic bias in a CV scan was mimicked by PE experiments, and TR-SERS during the PE experiments also displayed these stochastic CO vibrations. We found that PE experiments at low overpotentials result in gaseous CO formation (10% FE), which is 10-fold higher than after static application of the same potential (−0.35 V_RHE_). Furthermore, the TR-SERS and PE experiments showed that directly after the anodic pulses, the vibrational signals of stochastic CO and carbonate species are most intense, suggesting that the production of CO is highest directly after the anodic pulse is switched to the cathodic pulse on partially oxidized surfaces. We find spectroscopic evidence for the role of highly active copper carbonate hydroxide species, which are generated during the anodic pulse in low-overpotential CO_2_-to-CO activation. Our results showcase that the anodization of the surface CO2RR not only changes the catalyst surface but also creates an interplay between the electrolyte ions and oxidized surfaces, which opens up an alternative reaction route to the preferential formation of CO at low overpotentials.

## Supporting Information Available

Experimental section, cyclic voltammograms, SEM images, X-ray diffractograms, additional in situ Raman heatmaps during different CV cycles and PE experiments, Raman spectra of copper oxide, Raman spectrum of carbonate ions in solution, activity data for pulsed electrolysis, ECSA analysis, in situ Raman heatmaps in D_2_O and ^13^CO_2_, Raman spectra of stochastic carbonate and CO vibrations, in situ Raman spectra during PE in different concentrations of electrolyte, Raman spectra of malachite, and proposed reaction mechanism (PDF)
