# Supplementary material for: Probing the Dynamics of Low-Overpotential CO2-to-CO Activation on Copper Electrodes with Time-Resolved Raman Spectroscopy
Source: J Am Chem Soc. 2022 Aug 11;144(33):15047–58. doi: 10.1021/jacs.2c03172 (PMC9413204; doi:10.1021/jacs.2c03172)
Supplement: Supplementary file 1 — ja2c03172_si_001.pdf [file ja2c03172_si_001.pdf]

Supporting information

Probing the Dynamics of Low Overpotential CO_2-to-CO Activation on Copper Electrodes with Time-Resolved Raman Spectroscopy

Jim de Ruiter, Hongyu An, Longfei Wu, Zamorano Gijsberg, Shuang Yang, Thomas Hartman, Bert M. Weckhuysen, Ward van der Stam**

1. **Experimental Section**

Chemicals and materials

Copper foil (Cu, purity: 99.9999%, puratonic) was purchased from Alfa Aesar. Potassium bicarbonate ($KHCO_3$, purity >99.7%) was purchased from Fluka. Sulfuric acid (H_2SO_4, AnalaR NORMAPUR® analytical reagent, 95~97%) was purchased from VWR Chemicals. Carbon dioxide gas (CO_2, purity: 99.995%) was purchased from Linde Gas.

Catalyst preparation

99.9999% pure 0.1 mm copper foil was cut and sonicated for 5 min in 0.1 M HNO_3. The foil was washed with MQ H_2O and cupper dendrites were grown on top of the foil using chronopotentiometry with -500 mA for 25 s in an acidic $CuSO_4$ solution. This solution was prepared by dissolving 5 g of $CuSO_4$*5 H_2O in 90 mL MQ H_2O and 10 mL concentrated H_2SO_4 was added. The electrodeposited copper foil (CuED) was carefully washed again with MQ H_2O before use.

Electrochemical measurements

All electrochemical experiments were performed in a CO_2 saturated 0.1 M $KHCO_3$ solution (pH=6.8). A Pt mesh was used as a counter electrode and a leakless Ag/AgCl reference electrode (eDAQ) was used as a reference. The potential was converted to RHE using the following formula:

$$E(vs.\ RHE) = E(vs.\ Ag/AgCl) + 0.197\ V + 0.0591 * pH$$

Cyclic voltammetry (CV) measurements were performed using a Ivium compactstat.h10800 potentiostat. The cycles started at -0.1 V_{RHE} and ranged between +1.0 to -0.85 V_{RHE} using 10 mV step size and a scan rate of 10 mVs^{-1}. Pulsed electrolysis (PE) was performed by applying a cathodic potential of 50 s alternated by a 10 s anodic pulse of 1.0 V_{RHE}.

The activity evaluation of CO_2RR was carried out in a custom quartz H-cell. A platinum mesh was used as counter electrode, and a leakless Ag/AgCl reference electrode (eDAQ) was used for controlling the applied potential on the working electrode. The flow rate of CO_2 was kept at 10 sccm. CO was quantified using an Interscience online gas chromatography (GC) equipped with an FID detector. The GC injected a sample every 4 min. The Faradaic Efficiency (FE) was calculated using the following formula:

$$FE\ (\%) = \frac{c \cdot \mathrm{f} \cdot n_i}{i \cdot V_m \cdot 60\mathrm{sec/min} \cdot 1000000\mathrm{ppm}} \times 100\%$$

where c is the concentration obtained from the GC (in ppm), f is the flow rate of CO_2 gas (in mL/min), F is Faraday's constant (96485 C/Mol), n_i is the number of electrons transferred to a given product, i is the average current in 4 min (A), V_m is the volume of 1 mol gas at reaction temperature and pressure (24451 mL/mol).

Raman spectroscopy measurements

The Raman spectroscopy measurements were performed on a Renishaw InVia Raman microscope with a 785 laser. For time-resolved measurements we measure in static mode with a Nikon N40X-NIR water-dipping objective, 2.5 mW laser power and an acquisition time of 780 ms that, together with mechanistic time-delay (e.g., open and closing of the shutter) resulted in a time resolution of 1 spectrum per second. Time-resolved (TR) Raman spectroscopy measurements during cyclic voltammetry were carried out in the following way. To measure at 1 spectrum per second, while keeping reasonable Raman signal, we measured different Raman windows separately (230-650 cm^{-1}, 875-1245 cm^{-1}, 1285-1630 cm^{-1}, 1855-2160 cm^{-1}). In every Raman window, we measured 4 cycles and subsequently changed to a new Raman window to measure again 4 cycles until we measured all the Raman windows of interest. During the analysis of the data, we added the CV data and the different Raman windows together using the data of every 4th cycle. We confirmed that the induced current in every 4th cycle was always comparable to ensure that the Raman probed a similar surface. TR-Raman spectroscopy measurements during pulsed electrolysis were performed in a similar way. We measured CO between 1900-2100 cm^{-1}, and Cu_2O between 250-700 using the same Raman parameters as described above. To capture a broader range in the PE experiments, we decreased the time resolution to ± 6 seconds to collect a range of 200-2200 cm^{-1}.

Grazing incidence X-ray diffraction measurements

X-ray diffraction (XRD) measurements were performed on a Bruker DISCOVER d8 with Cu Kα (1.540 Å) radiation using a grazing incidence mode. An angle of incidence of 0.3 degrees to ensure surface sensitivity.

Scanning electron microscopy (SEM)

SEM images were acquired on a FEI Helios Nanolab G3 with accelerating voltage of 5.0 keV and probe current of 0.2 nA.

2. Additional Experimental Data

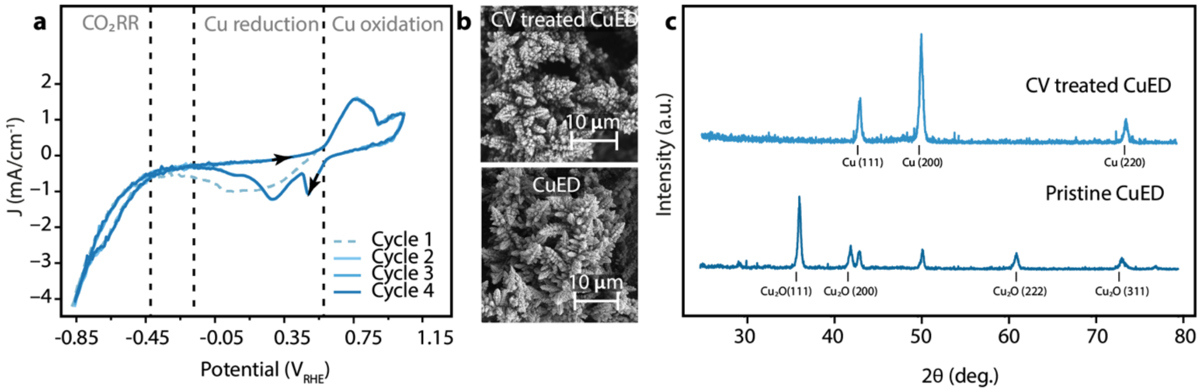

Figure SI 1. (a) Cyclic voltammogram (CV) plot of electrodeposited copper (CuED) in CO_2 saturated 0.1M $KHCO_3$ electrolyte entailing the copper reduction (+0.55 to –0.15 V_{RHE}), oxidation (>+0.55 V) and CO2RR potential windows (<–0.45 V). (b) Scanning electron microscopy (SEM) images of pristine CuED (bottom) and CV-treated CuED (top) showing dendrite Cu-crystals. (c) grazing incidence-X-ray diffraction (GI-XRD) patterns of pristine (bottom) and CV treated (top) CuED showing surface oxide removal after CV treatment.

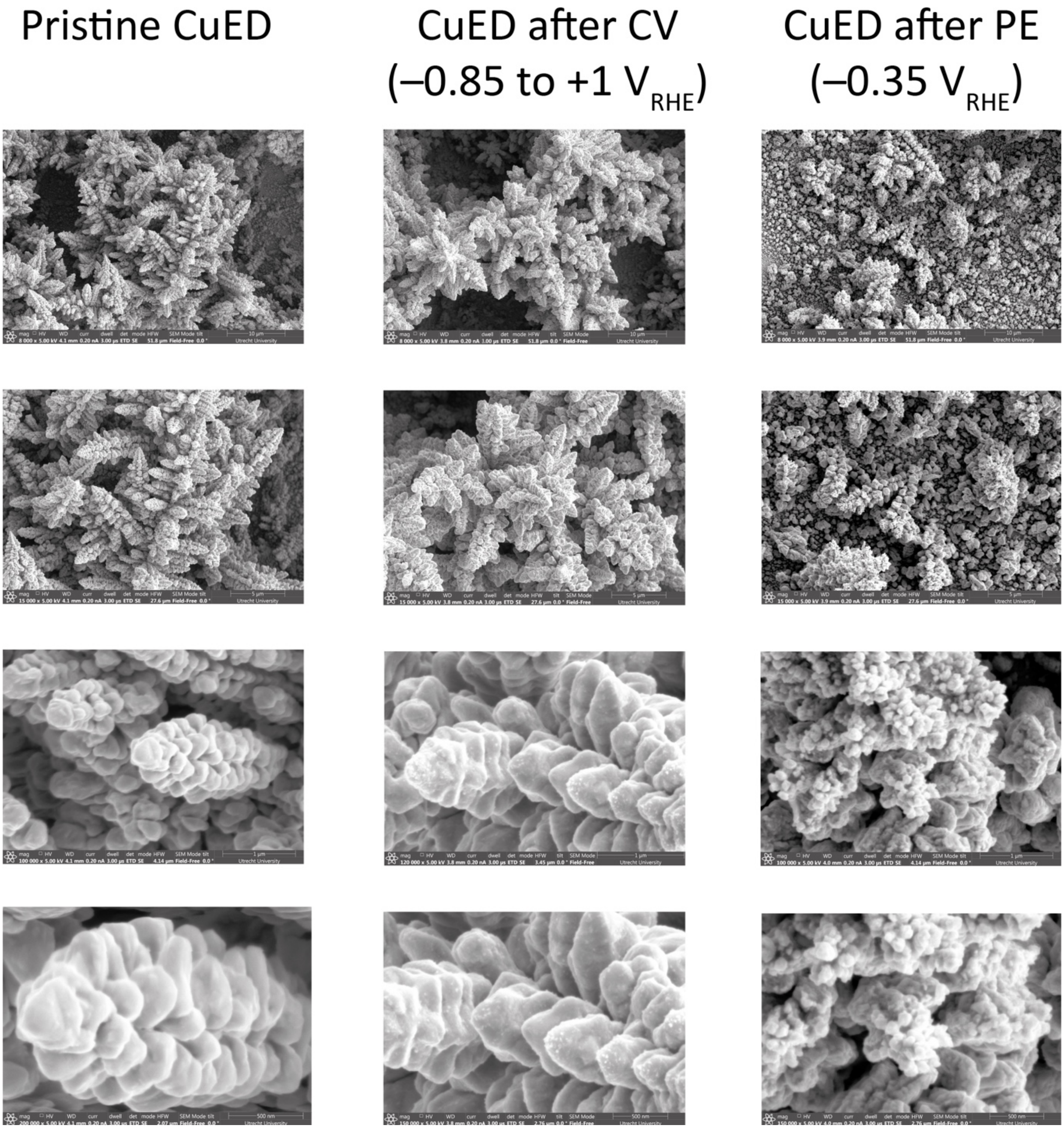

Figure SI 2. Scanning electron microscopy (SEM) images of, **left**: the pristine electrodeposited copper (CuED), **middle**: CuED after 4 cyclovoltammetry (CV) cycles and **right**: CuED after pulsed electrolysis (PE) at 150 s at –0.35 V_{RHE} and 10 s at 1 V_{RHE}.

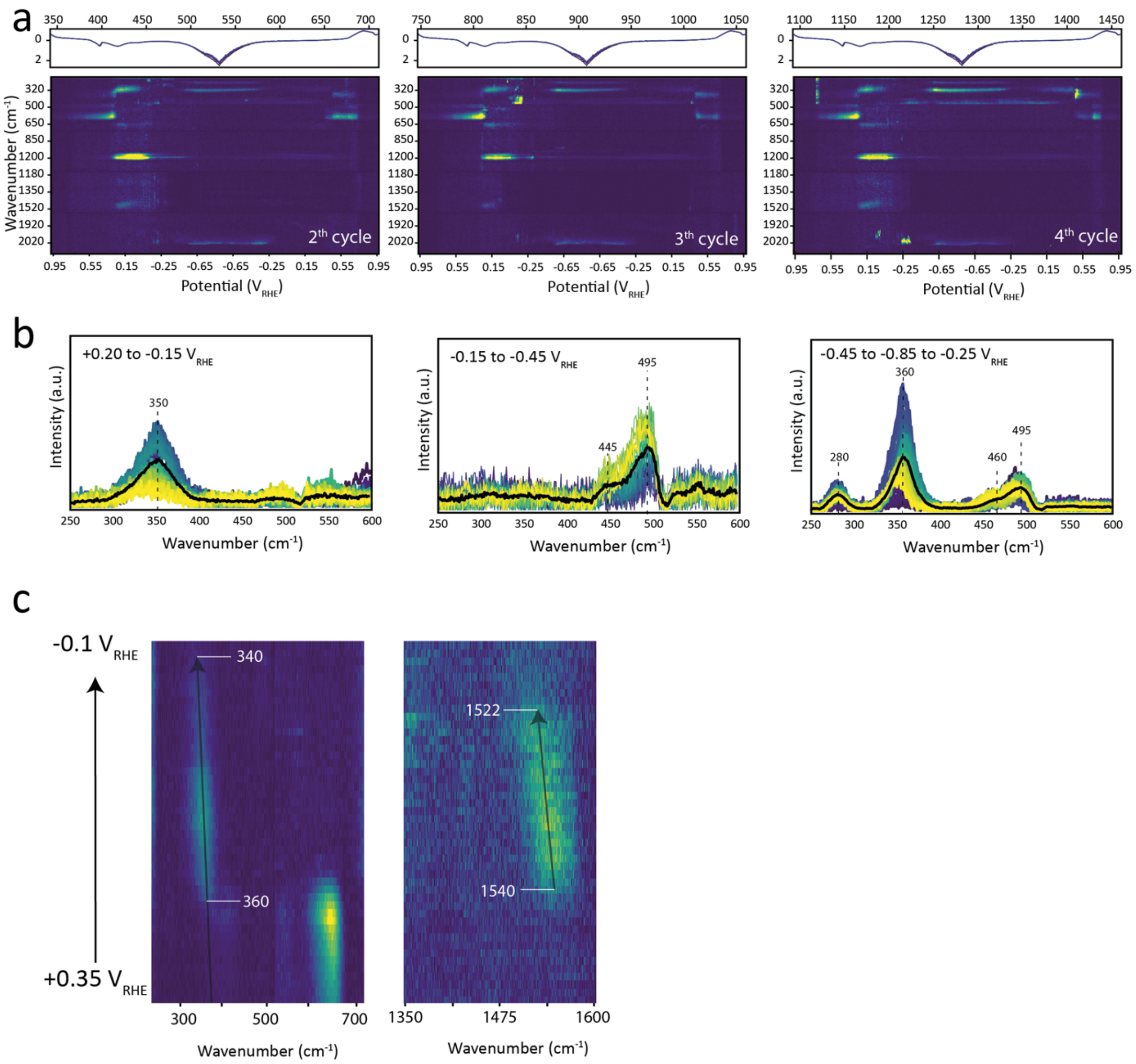

Figure SI 3. a) Time-resolved surface-enhanced Raman spectroscopy (TR-SERS) heatmaps of the 2nd, 3rd and 4th cyclic voltammetry (CV) cycles showing repetition of the obtained data. b) Some 2-D plots potentials windows corresponding to the heatmap of the 4th cycle. The black lines represent the average spectra of all the colored spectra in the panel. c) Zoom-in of the area between 0.35 and –0.1 V_{RHE} of the 4th CV cycle showing vibrational shift for the 350 and 1540 cm^{-1} Raman bands as a function of the potential applied.

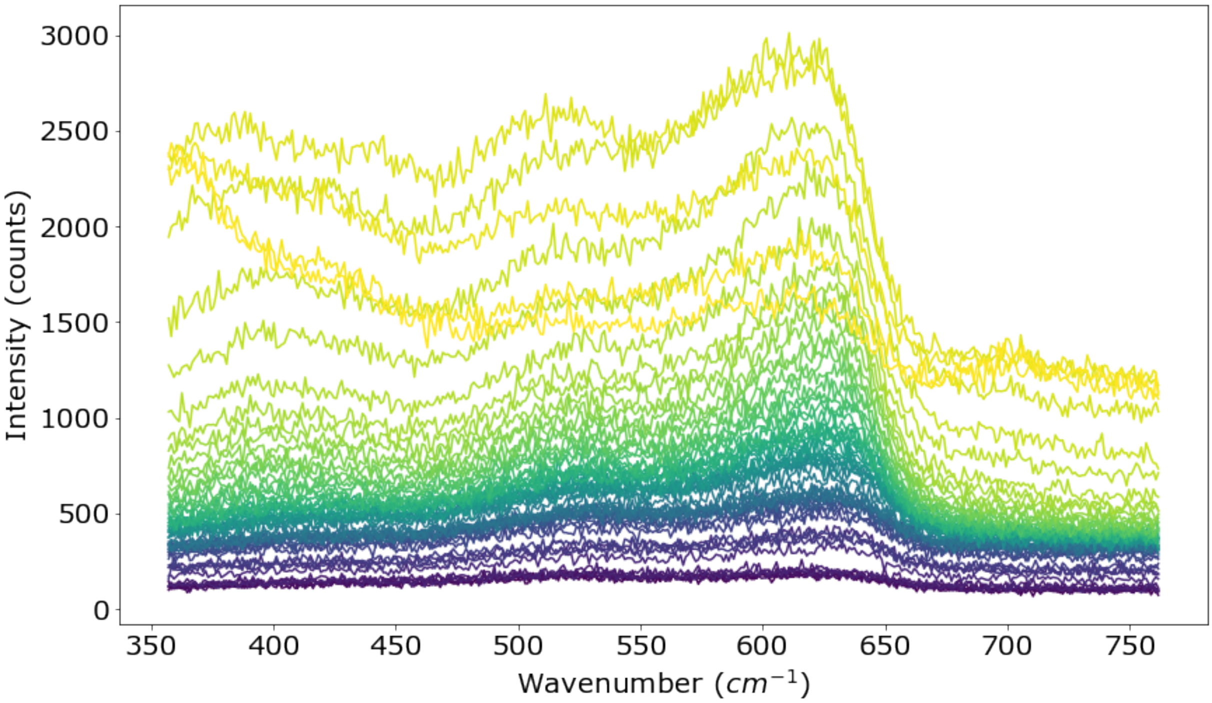

Figure SI 4. Evolution of Cu_2O in the 4th CV cycle from +0.55 (purple) to +0.25 V_{RHE}. The shape of the spectra change overtime revealing changes in the Cu_2O lattice.

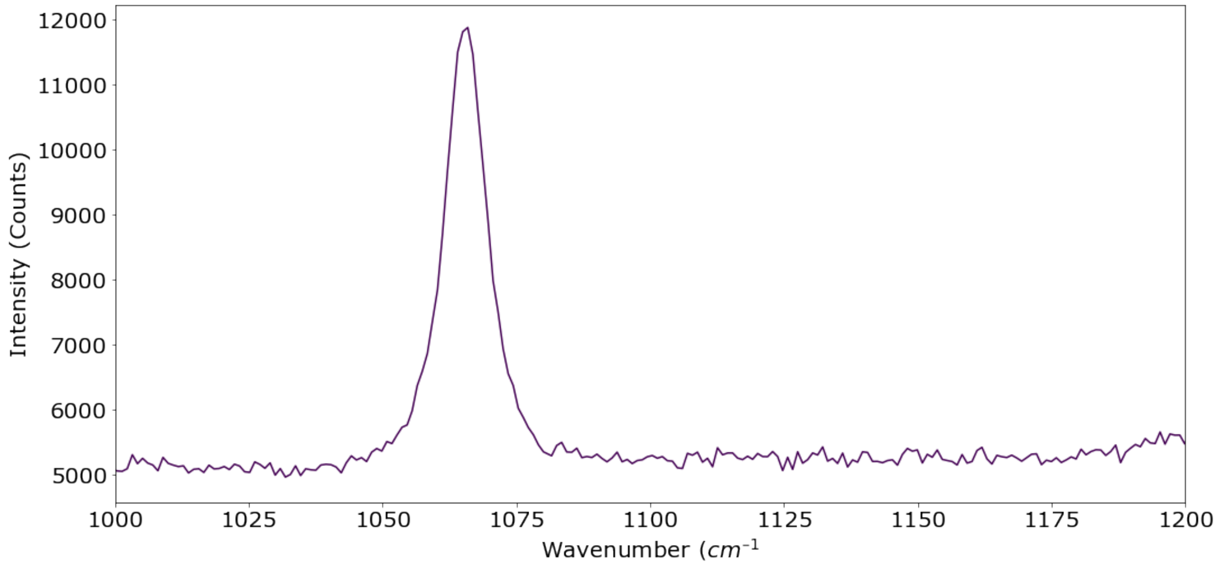

Figure SI 5. Raman spectrum of carbonate ($KHCO_3$) in solution, pH = 11.4. pH was adjusted by adding drops of 5M KOH adding to 0.1M $KHCO_3$. Peak is assigned to the symmetric stretching mode of carbonate.

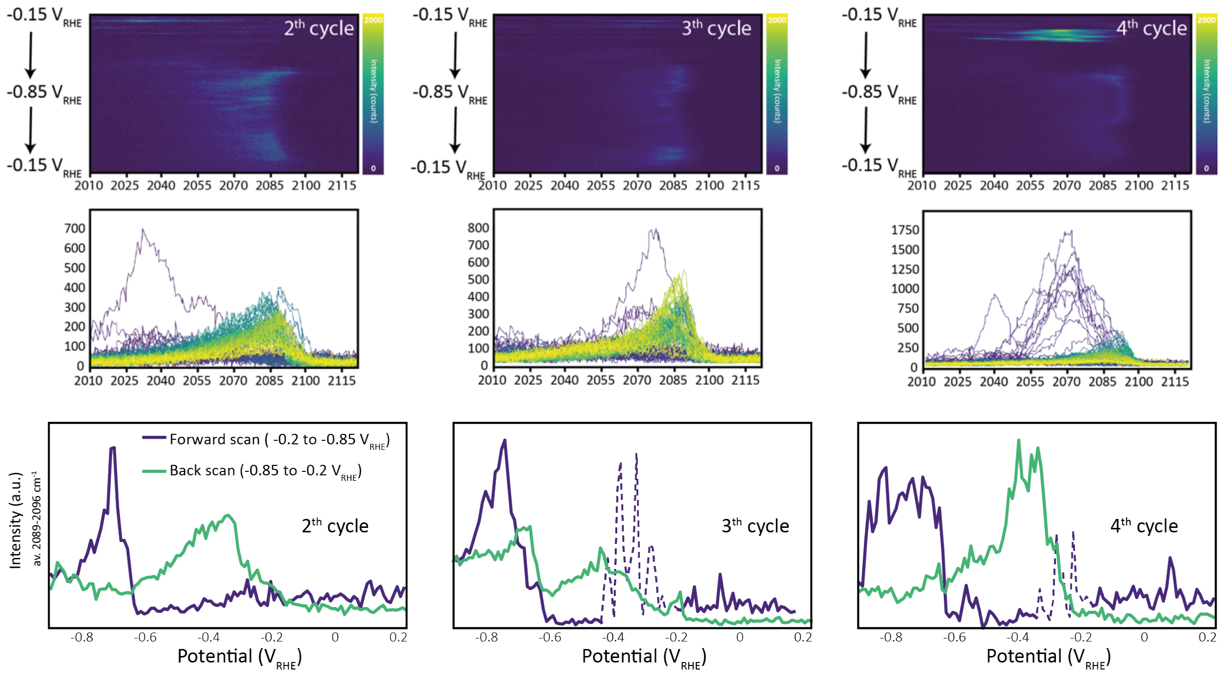

Figure SI 6. Heatmaps of the time-resolved surface enhanced Raman spectroscopy (TR-SERS) data during cyclovoltammetry (CV) measurements at cycles 2,3 and 4 between –0.2 to –0.85 V_{RHE} (forward scan) and back from –0.85 to –0.2 V_{RHE} (backward scan). In the forward scan, between 0 and –0.4 V_{RHE}, a different Cu-CO species is observed compared to the subsequent Cu-CO species. The 2-D plots below show the same data). If we take the maxima of the CO vibrations between 2089 and 2096 cm^{-1} in this potential window and plot the average vibrational energy against the potential, a hysteresis profile for CO adsorption/desorption is obtained

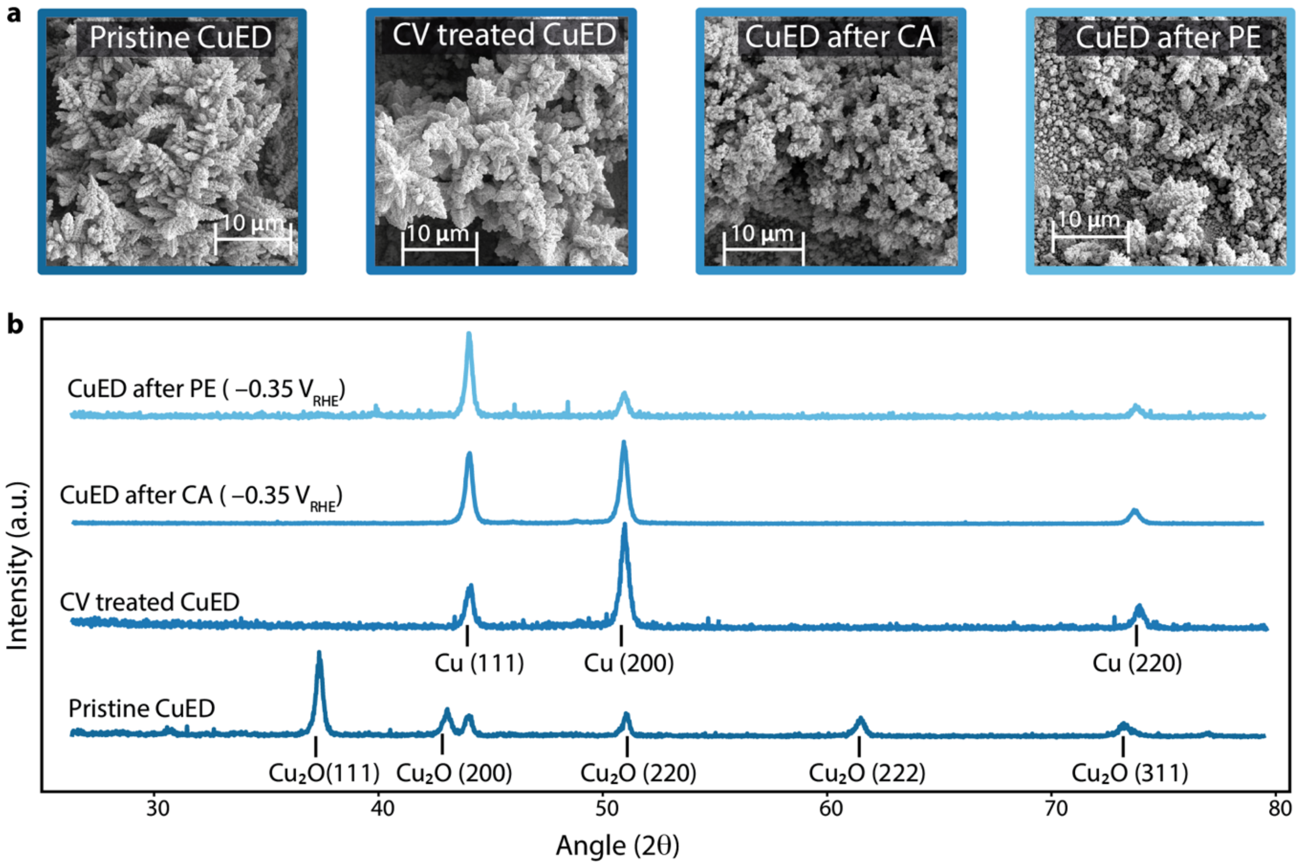

Figure SI 7. (a) Scanning electron microscopy (SEM) images of pristine electrodeposited copper (CuED), cyclovoltammetry (CV) treated CuED, CuED after chrono-amperometry (CA) and pulsed electrolysis (PE). (b) X-ray diffraction (XRD) patterns of the same materials.

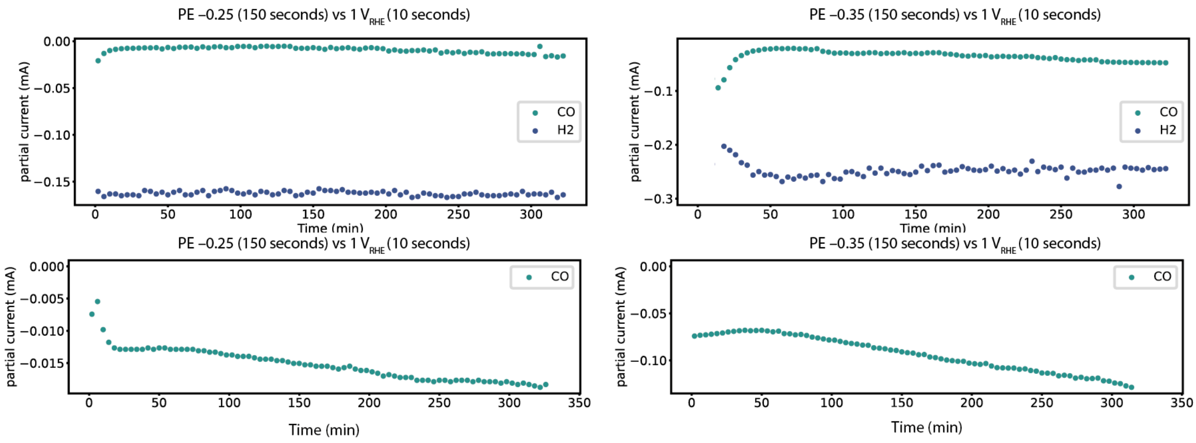

Figure SI 8. Partial current densities in PE experiments. Top (left and right respectively): Partial current for CO and H_2 at –0.25 V_{RHE} and –0.35 V_{RHE}. Bottom (left and right respectively): Partial current for CO at –0.25 V_{RHE} and –0.35 V_{RHE} corresponding to the activity values in Figure 2 of the main text and Figure SI 8 and 9. Hydrogen was not measured in this experiment. All experiments show the increase in CO over time. Formate could not be detected with ^{1}H-NMR.

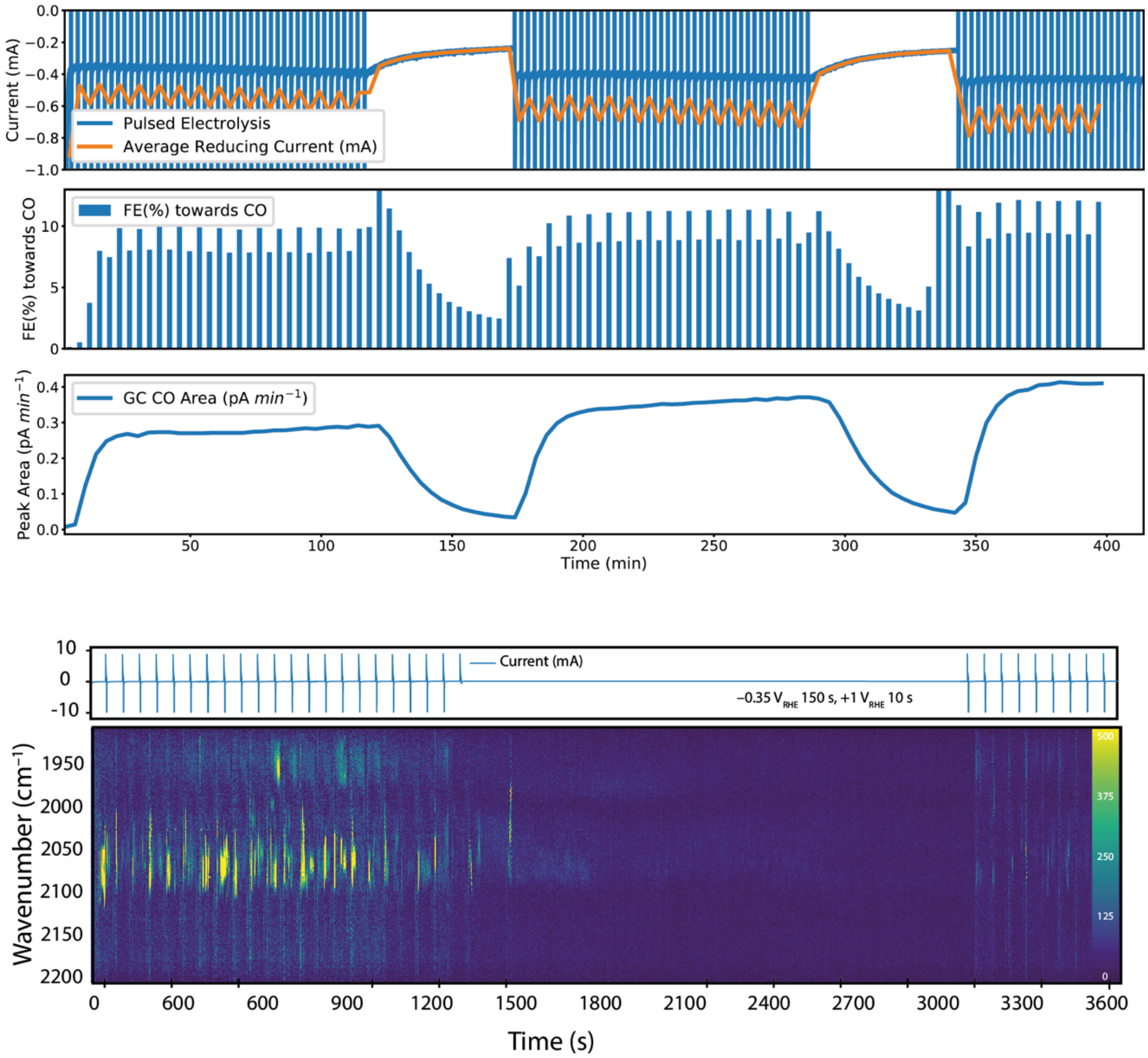

Figure SI 9 – top: Overview of activity measurements combining pulsed electrolysis with chronoamperometry. The FE towards CO drastically decreases when the anodic pulses were not present. Re-introducing the anodic pulses increases the activity to CO again. With TR-SERS we can see that under CA, no stochastic CO vibrations are detected, while during PE, these are abundantly present.

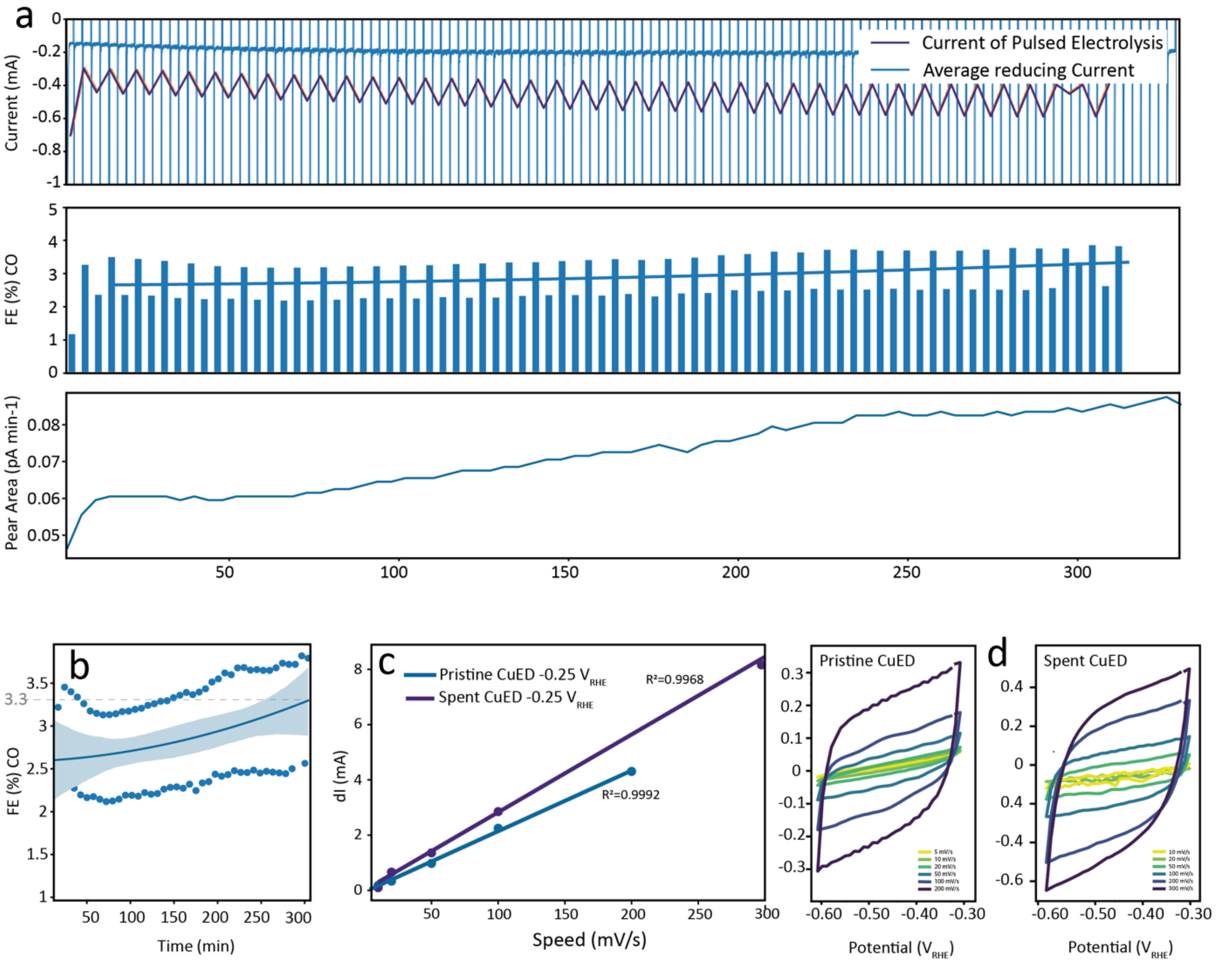

Figure SI 10. Overview of the activation experiments in pulsed electrolysis (PE) at 150 s –0.25 V_{RHE}, 10 s 1 V_{RHE}. a) Potentiostat (upper) and gas chromatograph (GC) (bottom) output showing an increase in the current and CO production over time. The GC took a sample every 4 min. The purple line is the average reduction current over 4 min and was used to calculate the Faradaic efficiency (FE) of each GC injection. Due to the introduction of anodic pulses, high cathodic current is induced directly after an anodic pulse causing the average reduction current to ‘zigzag’ which subsequently creates the same pattern for the FE calculations. A second order regression fit was used to optically fit the data, showing an increase of FE during the experiment. The fit can be seen in panel b. b) Calculated FE activity using the average current, fitted with a second order regression line to average out the ‘zigzag’ motion. c) Electrochemical surface area fit showing an increase in electrochemical active surface area (ECSA) after the reaction. The data is obtained from the cyclic voltammetry (CV) experiments shown in panel d. d) CV data with varying scan speed (mVs^{-1}) on pristine and spent electrodeposited copper (CuED) used for ECSA measurements.

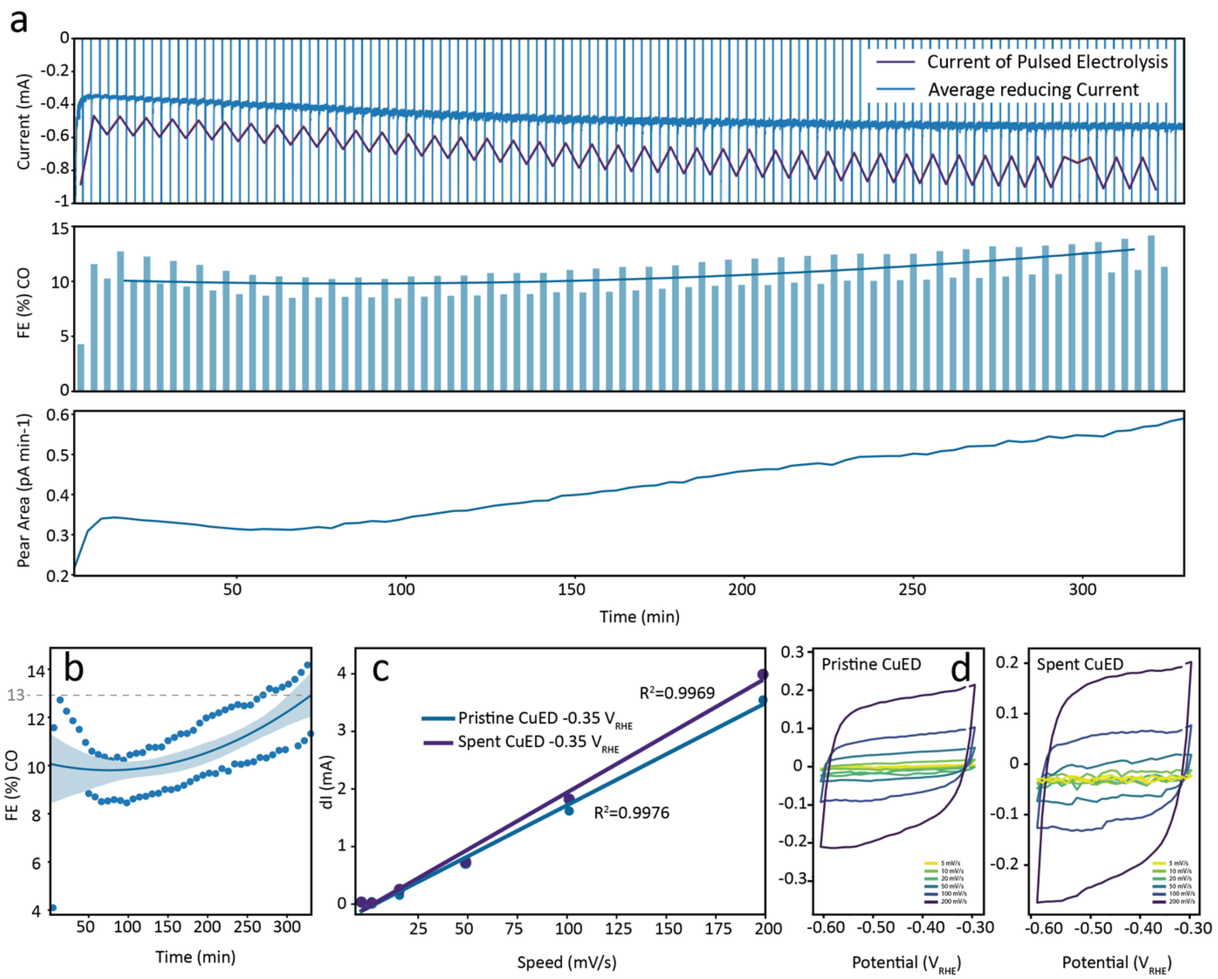

Figure SI 11. Overview of the activation experiments in pulsed electrolysis (PE) at 150 s –0.35 V_{RHE} and 10 s 1 V_{RHE}. a) Potentiostat (upper) and gas chromatography (GC) (bottom) output showing an increase in current and CO production over time. The GC took a sample every 4 min. The purple line is the average reduction current over 4 min and was used to calculate the Faradaic efficiency (FE) of each GC injection. Due to the introduction of anodic pulses, high cathodic current is induced directly after an anodic pulse causing the average reduction current to 'zigzag' which subsequently creates the same pattern for the FE calculations. A second order regression fit was used to optically fit the data, showing an increase of FE during the experiment. The fit can be seen in panel b. b) Calculated FE activity using the average current, fitted with a second order regression line to average out the 'zigzag' motion. c) Electrochemical surface area fit showing an increase in the electrochemical active surface area (ECSA) after the reaction. The data is obtained from the cyclovoltammetry (CV) experiments shown in panel d. d) CV data with varying scan speed (mVs^{-1}) on pristine and spent electrodeposited (CuED) used for ECSA measurements.

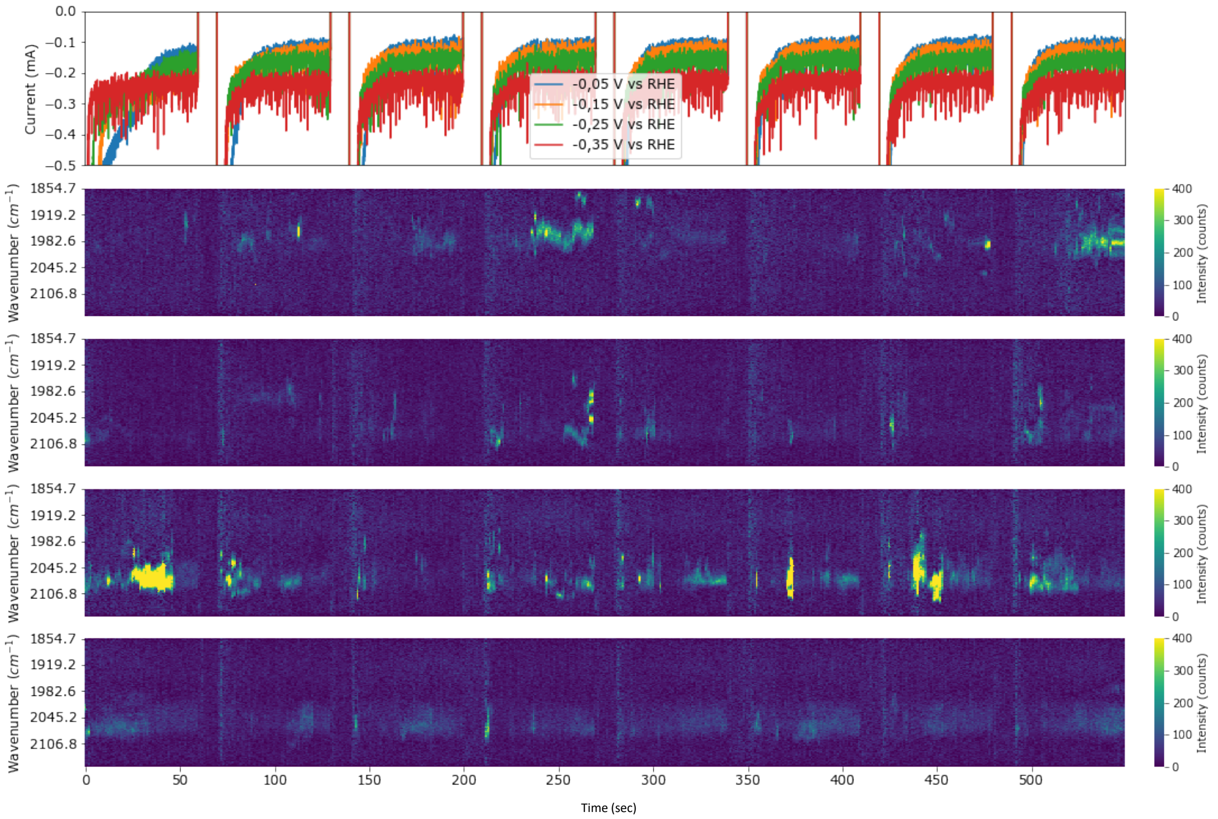

Figure SI 12. Heatmaps showing separate pulsed electrolysis experiments using (top to bottom); –0.05, –0.15, –0.25 and –0.35 V_{RHE} for 60 seconds alternated by 10 seconds of 1 V_{RHE}. In all experiments, the random CO is observed but only at –0.35 V_{RHE}, the static CO vibrations at 2050-2090 are visible at longer timescales. On top, a zoom in of the cathodic current is shown, displaying increased current with increasing cathodic potential.

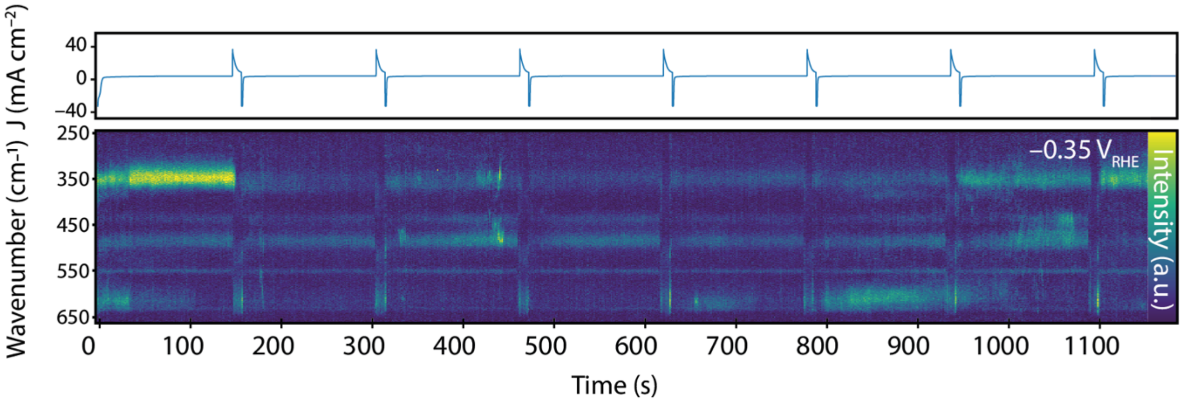

Figure SI 13. TR-SERS in the low Raman window (250-650 cm^{-1}) during PE at –0.35 V_{RHE}. Intensity is plotted in a heatmap, as function of time. PE data is positioned above the heatmap to overlap with the Raman data. During the anodic pulse, the vibrations of Cu_2O are observed. During the cathodic potentials, many bands are observed which requires further research. The presence of the 630 cm^{-1} band during the cathodic pulse indicated Cu_2O could be present under reductive currents.

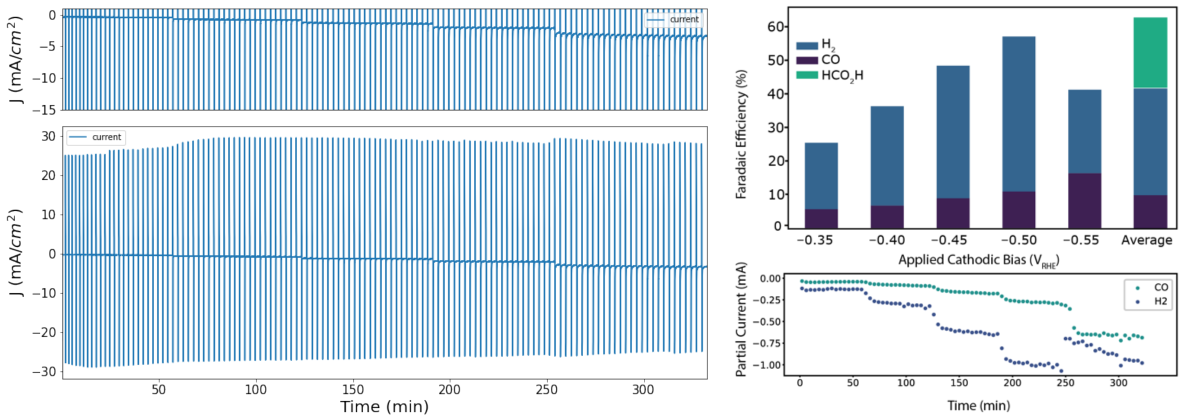

Figure SI 14. Left: Current data in the pulsed electrolysis experiment using –0.35, –0.40, –0.45, –0.50 and –0.55 V_{RHE} as cathodic potentials for 150 seconds alternated by 1 V_{RHE} for 10 seconds. The five potential windows can be distinguished by the decrease in reducing current each 66 minutes. Top graph is a zoom-in of the static chronoamperometry current Right: Activity bar plot showing the average product distribution of each cathodic potential windows during the pulsed electrolysis experiments. The total cathodic current was used to calculate the Faradaic efficiency. The current that is induced by the re-reduction of the surface after an oxidative pulse was not included in the calculation and explains the lack of FE (in other words, the FE does not add up to 100%). Formate could only be detected by NMR after the end of the reactions and only reflects the average production for the entire pulse program.

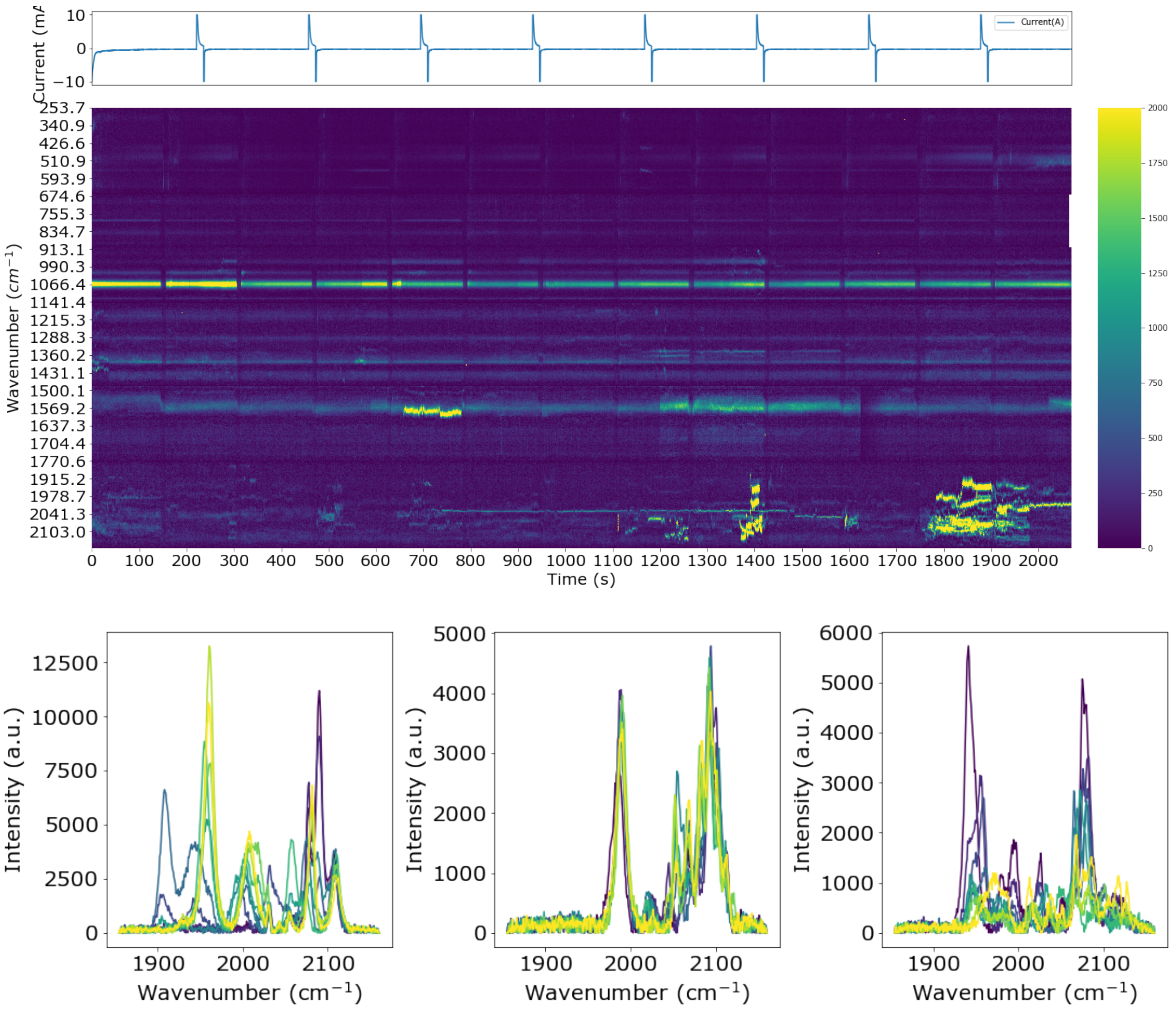

Figure SI 15. Heatmap showing a pulsed electrolysis experiment using –0.35 V_{RHE} for 150 seconds alternated by 10 seconds of 1 V_{RHE} in D_2O. The electrolyte was prepared by saturating a 0.1 M K_2CO_3 solution in D_2O with CO_2 till a pH value of 6.9 was reached. Multiple stochastic CO vibrations can be observed between 1900 and 2100 cm^{-1}. We should note that in D_2O, the surface is much less oxidized as D_2O is less oxidizing than H_2O. The surface remains relatively intact yielding very good SERS. This experiment illustrates that the observed vibrations do not correspond to Cu-H. For Cu-D, we would expect a significant change in vibrational energy from 2050 cm^{-1} to 1041 cm^{-1} (following the equation $\frac{v1}{\mu 1} = \frac{v2}{\mu 2}$, in which μ1 is 0.98 ($\frac{mCu*mH}{mCu+mH}$ with mCu = 63 u and mH = 1 u), and μ2 is 1.94). The three 2D spectra plots underneath the graphs correspond to the spectra collected at t = 1390-1400 s, 1800-1810 s and 1900-1910 s, respectively.

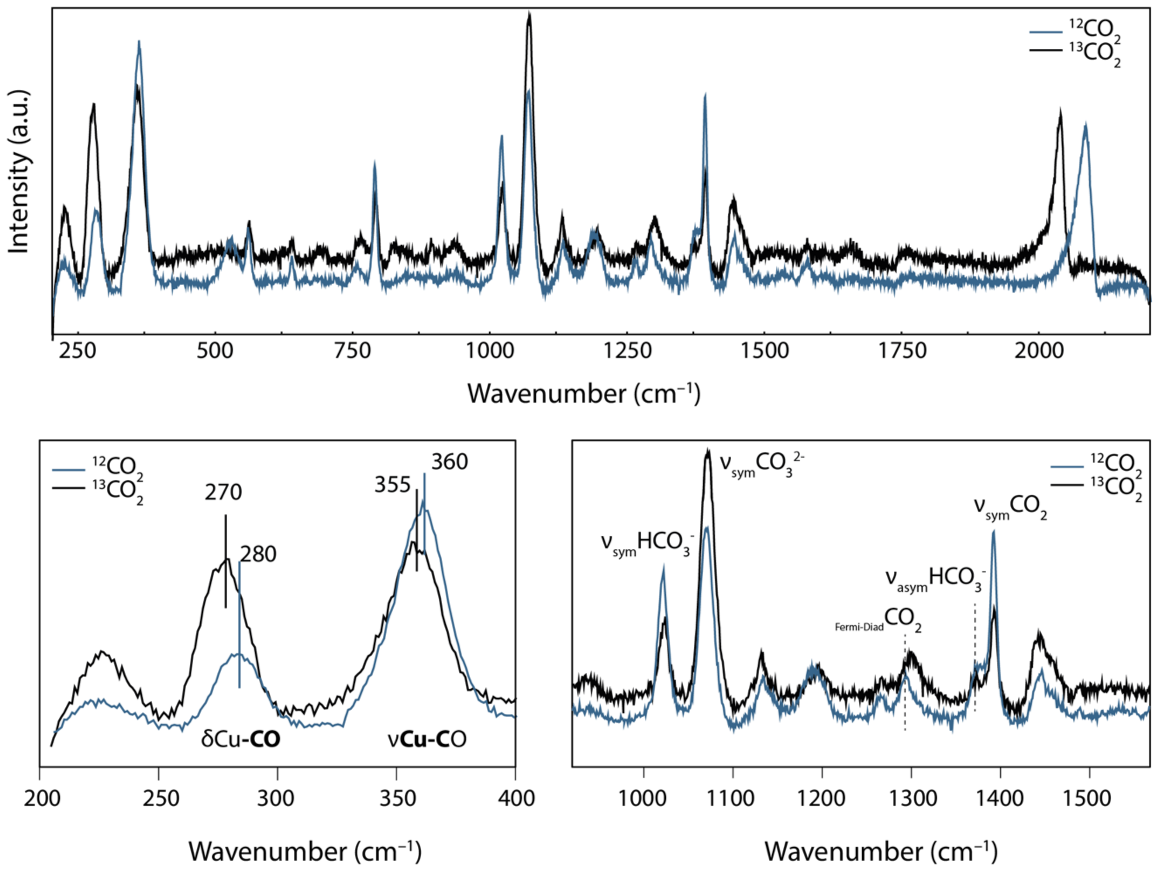

Figure SI 16. Average spectra of PE at –0.55 V_{RHE} (150 s, 10 sec +1 V_{RHE}) using $^{12}CO_2$ and $^{13}CO_2$. A clear shift for the CO intermediate is observed for the vibrations at 280, 360 and 2090 cm^{-1}. The peaks in the carbonate region (900-1600 cm^{-1}) are not influenced by the increased mass, since the vibrations are dominated by movement of the oxygen atoms. The references of the assignments can be found in Table SI 1.

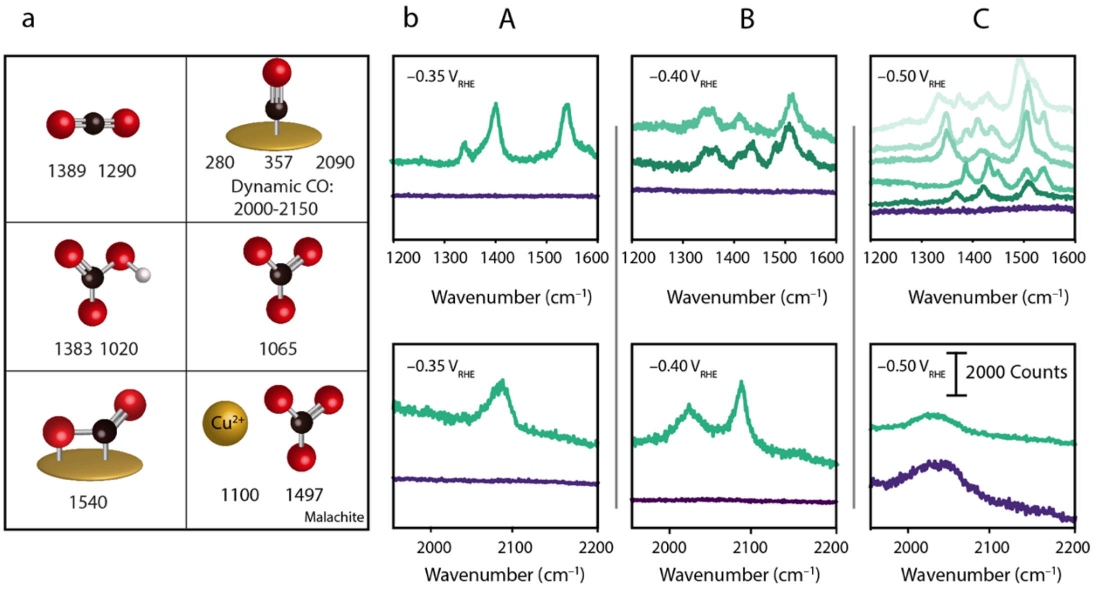

Figure SI 17. (a) Schematic overview of known vibrations of CO and carbonate related species[1–6]. An overview is provided in Table SI1. (b) Some zoom-in (non-baseline corrected) line plots showing the difference in stochastic behavior after (green) and before (purple) an anodic pulse. The moment in time of the line plots is indicated in Figure 4 with ‘A B and C’. In Figure SI 18, more spectra collected over a wider wavenumber range are shown.

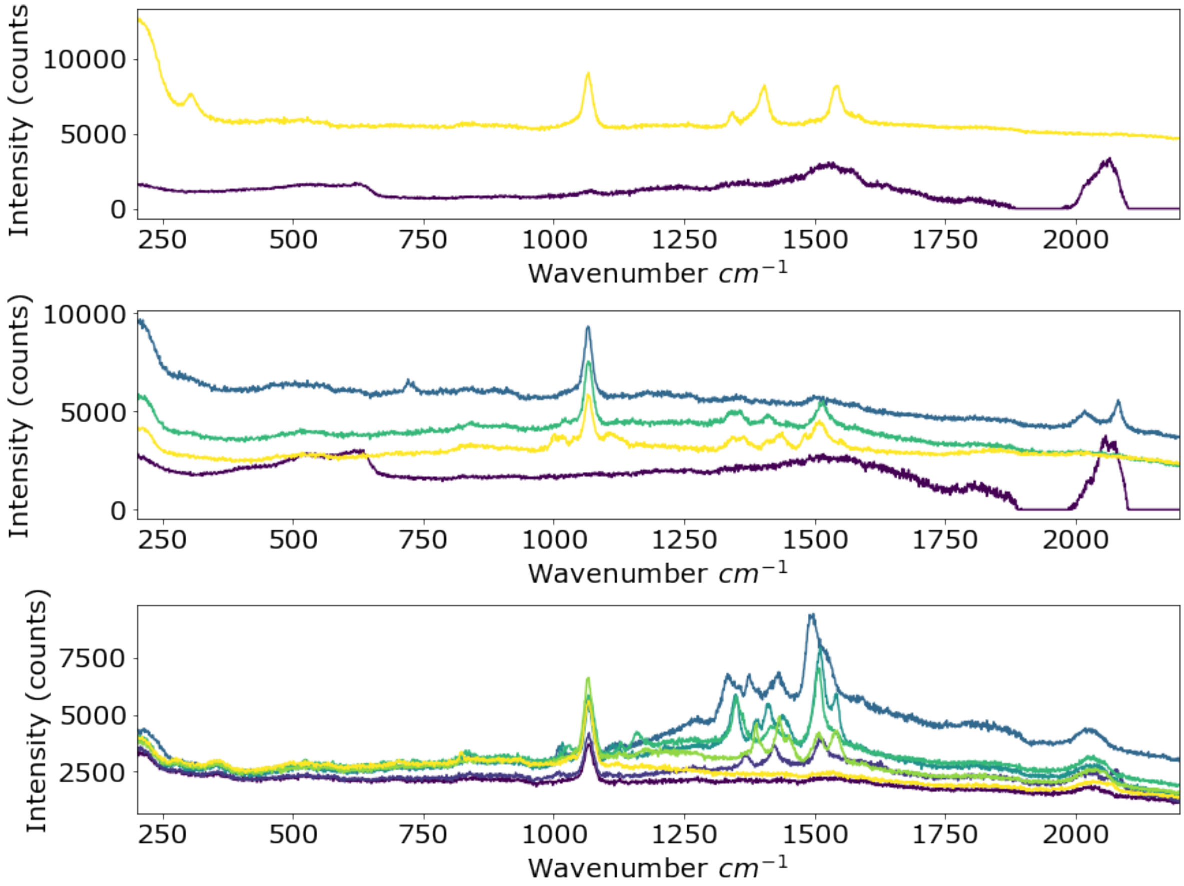

Figure SI 18. Raman spectra over the entire Raman shift region corresponding to the graphs in Figure SI 14. The stochastic vibrations corresponding to the A, B and C domains in Figure 4 in the main text. Cathodic potentials from top to bottom: –0.35, –0.40, –0.50 V_{RHE}.

Table SI 1 - Overview of vibrations observed in this work.

**Wavenumber (cm^{-1})**	**Species**	**Vibration**	**Reference**
400,520,620	Cu_2O (incl defects)	520: vibrational mode of Cu2O lattice,	[7,8]
280	Cu-CO	CO bend	[9–12]
360	Cu-CO	Cu-(CO) stretch	[9–12]
350, 1540	Adsorbed carbonate / carboxylate	Under debate	[4,5,13]
700, 1050, 1065, 1420	HCO_3^- and CO_3^{2-}	1050, 1070: Symmetric stretching of HCO_3^- and CO_3^{2-} respectively<br>700: $-CO_2^-$ bending<br>1420: antisymmetric stretching of CO_3^{2-}	[1–6,11,14]
1280, 1388	CO_2	Fermi-Diad mode CO_2,<br>Symmetric stretching of CO_2	[4]
2090	CO	CO stretch	[9–12]
2000-2100	Stochastic CO	CO stretch	This work

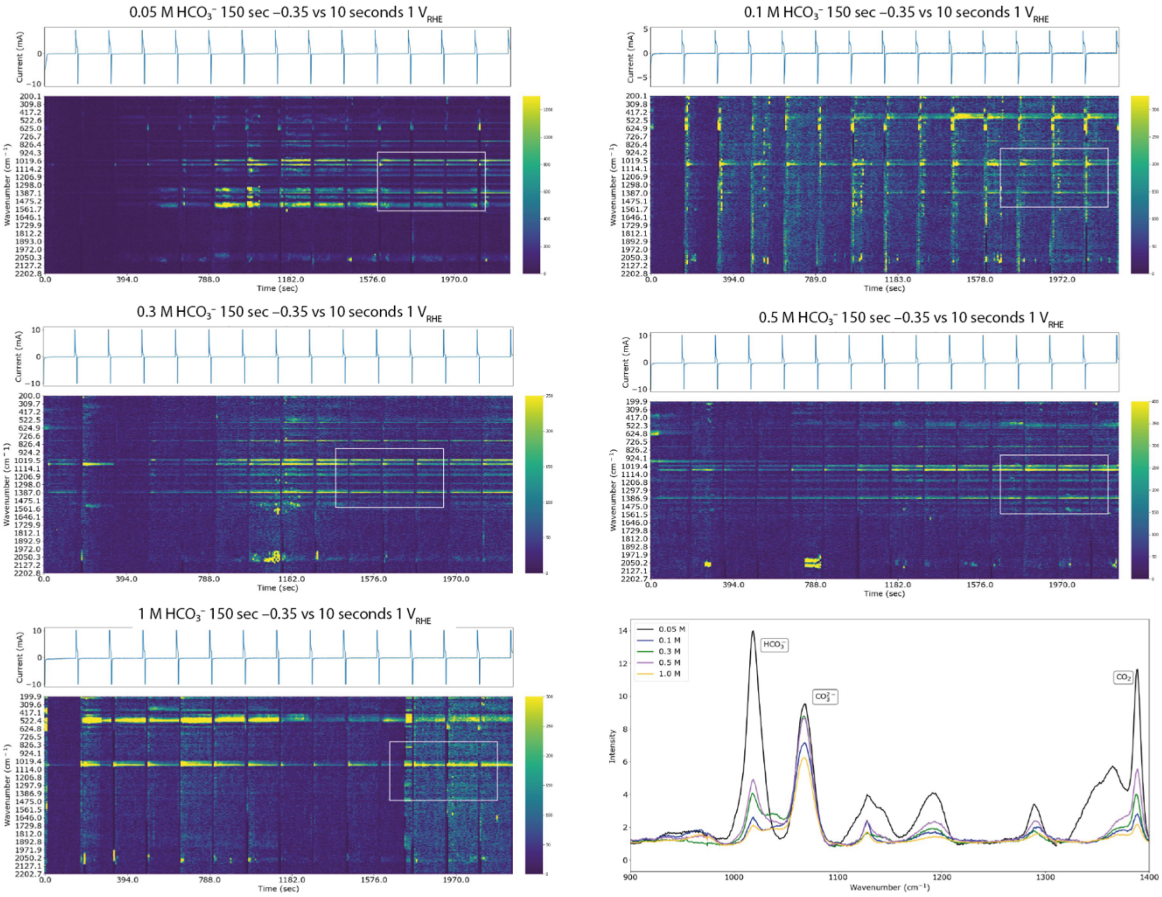

Figure SI 19. Pulsed electrolysis using –0.35 V_{RHE} for 150 seconds alternated by 10 seconds of +1 V_{RHE} in 0.05, 0.1, 0.3, 0.5, and 1 M CO_2 saturated HCO_3^-. The stochastic CO and carbonate vibrations are visible in each experiment. Right corner: Averaged Raman spectra of the electrolyte concentration PE experiments, normalized on the carbonate peak. An increase in HCO_3^- concentration results in an increase in local pH as evidenced by the reduction of the bicarbonate and CO_2 peak at 1035 cm^{-1} peak at 1388 cm^{-1}. Average taken in the area's indicated in the white squares of the heatmaps.

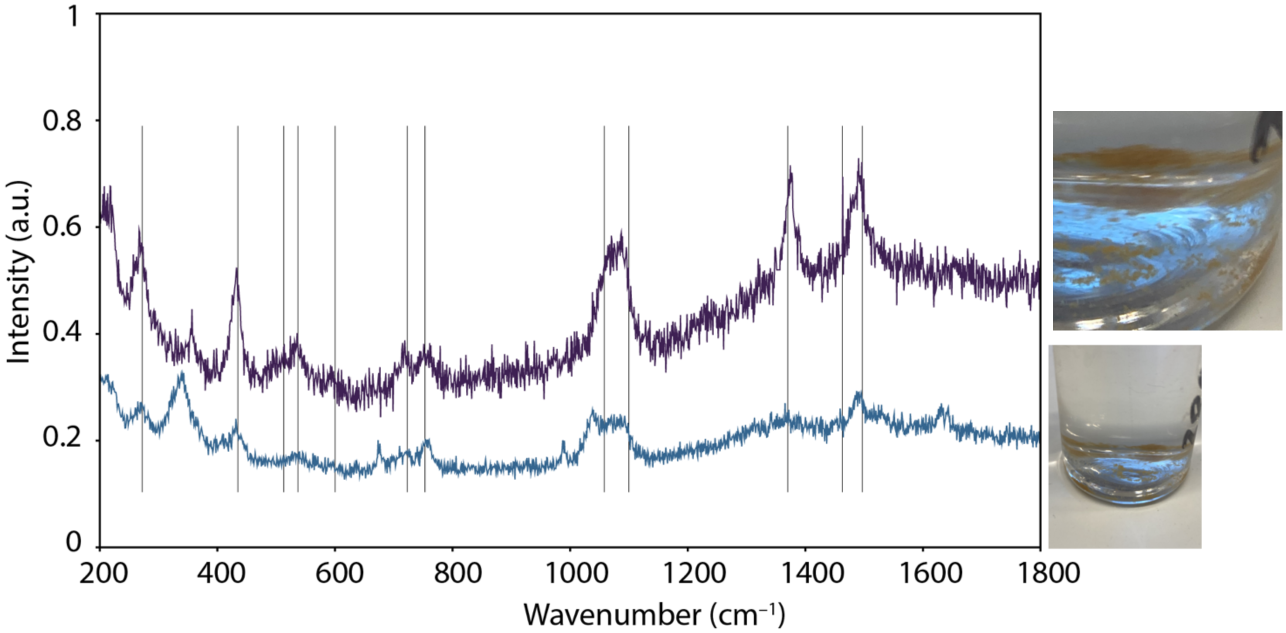

Figure SI 20. Raman spectra of the precipitates that were collected after the PE program. The gray lines indicate the wavenumber positions of malachite, according to[6].

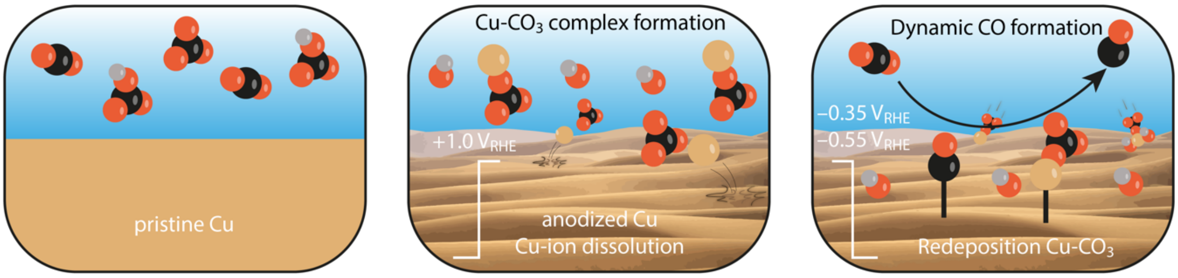

Figure SI 21. Proposed alternative reaction mechanism for the enhanced activity for CO production in CO2RR using pulsed electrolysis at low overpotentials. During the anodization, copper ions deplete into the electrolyte and coordinate to the carbonate ions forming various Cu-CO_3 complexes. With the increased local pH, these complexes can result in the formation of malachite (copper-carbonate-hydroxide), which was detected with Raman spectroscopy in the deposits that were observed after each experiment (Figure SI 19). When cathodic potentials were applied again, these copper-carbonate complexes can adsorb on the surface and be reduced directly to CO (at lower cathodic biases). At higher cathodic potentials (up to –0.55 V_{RHE}), the formation of the more static Cu-CO intermediates as well as the presence of the stochastic CO were observed, suggesting that the introduction of the anodic pulses induces a different reaction pathway for CO.

References

(1) Klingan, K.; Kottakkat, T.; Jovanov, Z. P.; Jiang, S.; Pasquini, C.; Scholten, F.; Kubella, P.; Bergmann, A.; Roldan Cuenya, B.; Roth, C.; Dau, H. Reactivity Determinants in Electrodeposited Cu Foams for Electrochemical CO_2 Reduction. *ChemSusChem* **2018**, *11*, 3449–3459.

(2) King, H. E.; Geisler, T. Tracing Mineral Reactions Using Confocal Raman Spectroscopy. *Minerals* **2018**, *8*, 158.

(3) Li, H.; Wei, P.; Gao, D.; Wang, G. In Situ Raman Spectroscopy Studies for Electrochemical CO_2 Reduction over Cu Catalysts. *Current Opinion in Green and Sustainable Chemistry* **2022**, *34*, 100589.

(4) Anderson, G. R. The Raman Spectra of Carbon Dioxide in Liquid H_2O and D_2O. *Journal of Physical Chemistry* **1976**, *81*, 273–276.

(5) Chernyshova, I. V.; Somasundaran, P.; Ponnurangam, S. On the Origin of the Elusive First Intermediate of CO_2 Electroreduction. *Proceedings of the National Academy of Sciences of the United States of America* **2018**, *115*, E9261–E9270.

(6) Mattei, E.; de Vivo, G.; de Santis, A.; Gaetani, C.; Pelosi, C.; Santamaria, U. Raman Spectroscopic Analysis of Azurite Blackening. *Journal of Raman Spectroscopy*; **2008**, 39, 302–306.

(7) Debbichi, L.; Marco De Lucas, M. C.; Pierson, J. F.; Krüger, P. Vibrational Properties of CuO and Cu_4O_3 from First-Principles Calculations, and Raman and Infrared Spectroscopy. *Journal of Physical Chemistry C* **2012**, *116*, 10232–10237.

(8) Singhal, A.; Pai, M. R.; Rao, R.; Pillai, K. T.; Lieberwirth, I.; Tyagi, A. K. Copper(I) Oxide Nanocrystals - One Step Synthesis, Characterization, Formation Mechanism, and Photocatalytic Properties. *European Journal of Inorganic Chemistry* **2013**, *2013*, 2640–2651.

(9) Zhan, C.; Dattila, F.; Rettenmaier, C.; Bergmann, A.; Kühl, S.; García-Muelas, R.; López, N.; Cuenya, B. R. Revealing the CO Coverage-Driven C–C Coupling Mechanism for Electrochemical CO_2 Reduction on Cu_2O Nanocubes via Operando Raman Spectroscopy. *ACS Catalysis* **2021**, *11*, 7694–7701.

(10) Gunathunge, C. M.; Li, X.; Li, J.; Hicks, R. P.; Ovalle, V. J.; Waegele, M. M. Spectroscopic Observation of Reversible Surface Reconstruction of Copper Electrodes under CO_2 Reduction. *Journal of Physical Chemistry C* **2017**, *121*, 12337–12344.

(11) Jiang, S.; Klingan, K.; Pasquini, C.; Dau, H. New Aspects of Operando Raman Spectroscopy Applied to Electrochemical CO_2 Reduction on Cu Foams. *Journal of Chemical Physics* **2019**, *150*, 041718.

(12) Akemann, W.; Otto, A. Vibrational Modes of CO Adsorbed on Disordered Copper Films. *Journal of Raman Spectroscopy* **1991**, *22*, 797–803.

(13) Moradzaman, M.; Mul, G. In Situ Raman Study of Potential-Dependent Surface Adsorbed Carbonate, CO, OH, and C Species on Cu Electrodes During Electrochemical Reduction of CO_2. *ChemElectroChem* **2021**, *8*, 1478–1485.

(14) Buzgar, N.; Apopei, A. I. The Raman Study of Certain Carbonates. *Geologie, Tomul* **2009**, *2*, 97–112.
